# Experimental Models to Study the Functions of the Blood–Brain Barrier

**DOI:** 10.3390/bioengineering10050519

**Published:** 2023-04-25

**Authors:** Andrzej Łach, Agnieszka Wnuk, Anna Katarzyna Wójtowicz

**Affiliations:** 1 Laboratory of Neuropharmacology and Epigenetics, Department of Pharmacology, Maj Institute of Pharmacology, Polish Academy of Sciences, 31-343 Kraków, Poland; lach@if-pan.krakow.pl (A.Ł.);; 2 Department of Nutrition, Animal Biotechnology and Fisheries, Faculty of Animal Sciences, University of Agriculture, 30-059 Kraków, Poland

**Keywords:** blood–brain barrier, *in vitro* modeling, permeability, HTS, TEER

## Abstract

The purpose of this paper was to discuss the achievements of in vitro modeling in terms of the blood–brain barrier [BBB] and to create a clear overview of this research area, which is useful in research planning. The text was divided into three main parts. The first part describes the BBB as a functional structure, its constitution, cellular and noncellular components, mechanisms of functioning and importance for the central nervous system, in terms of both protection and nourishment. The second part is an overview of parameters important in terms of establishing and maintaining a barrier phenotype that allows for formulating criteria of evaluation of the BBB *in vitro* models. The third and last part discusses certain techniques for developing the BBB *in vitro* models. It describes subsequent research approaches and models, as they underwent change alongside technological advancement. On the one hand, we discuss possibilities and limitations of different research approaches: primary cultures vs. cell lines and monocultures vs. multicultures. On the other hand, we review advantages and disadvantages of specific models, such as models-on-a-chip, 3D models or microfluidic models. We not only attempt to state the usefulness of specific models in different kinds of research on the BBB but also emphasize the significance of this area of research for advancement of neuroscience and the pharmaceutical industry.

## 1. Introduction

The blood–brain barrier (BBB) is a complex structure present in mammalian organisms and is responsible for maintaining the parameters of the internal environment of the central nervous system (CNS). At the same time, it participates in the delivery of nutrients to CNS cells, removal of their metabolites and gas exchange. Finally, it protects the CNS from the harmful influence of a variety of compounds. It is important to note a certain kind of dualism presented by the BBB—it is both a barrier and transport structure. Barrier function is implemented on three planes: a physical barrier made up of endothelial cells [ECs] and tight junctions [TJs] that bond them, a metabolic barrier formed by specific enzymes produced by BBB components, and a transport barrier, realized by a variety of transporting proteins that remove certain substances from the territory of the CNS [1]. Transport function is based on a few paths of penetration of the BBB by specific compounds, and these paths will be further reviewed later. It is commonly known that both described functions of this structure are impaired in cases of neurodevelopmental and neurodegenerative disorders, such as Alzheimer’s Disease and amyotrophic lateral sclerosis [2,3]. According to recent studies, the loss of integrity of the BBB is a cause of neurodevelopmental disorders including autism spectrum disorder (ASD) [4] or schizophrenia [5]. Considering the importance of this structure, reliable models of BBB are urgently needed for both clinicians and researchers.

This crucial role of the BBB in nervous system functioning, and thus for the entire organism, have made this structure an object of numerous studies leading to an understanding of its properties. A purely clinical approach also sets a demand for such studies, since the BBB both forms an obstacle for various substances that could potentially be used in neurological disease therapy and is impaired in the progression of many diseases [6]. Juxtaposition of the demand for profound understanding of the BBB structure and function with increasing popularity and advancement of *in vitro* methods sets an obvious direction of development for this branch of neurobiology. To date, many models have been established to mimic the BBB. They are intended to reflect aspects of the BBB that previously demanded *in vivo* testing, but more importantly, they offer an opportunity to conduct studies in a rigorous, repeatable manner that allows for the comparison of results between independent research teams.

As stated above, the BBB poses an obstacle for CNS therapies based on biologically active compounds that need to reach a certain concentration inside the CNS to induce pharmacological effects. Employing a purely chemical approach, it is possible to quickly design candidates for CNS-targeting drugs, but in reality, 98% of these candidates are unable to cross the BBB [6]. An obvious necessity arises for eliminating this percentage of compounds in the early stages of testing. Among others, this need for high-throughput screening translates to the increasing popularity of *in vitro* methods. *In vivo* techniques, while still used, have proven to be insufficient due to their high cost, time-consumption and low throughput as well as the moral dilemmas they create.

The purpose of the present work is to review, analyze and evaluate techniques and approaches to BBB *in vitro* modeling. First, we focus on describing the barrier’s composition and functioning, then we discuss the parameters important in terms of establishing and maintaining a barrier phenotype that allows for formulating criteria of evaluation of the BBB *in vitro* models. Finally, we discuss the possibilities and limitations of particular models.

## 2. Structure and Importance of the Blood–Brain Barrier

To discuss models employed in BBB research, it is crucial to first understand the barrier itself and its structure, characteristics and dependencies linking its components. The following section was devoted to these issues, partially creating a foundation for the later evaluation of certain models.

## 3. Endothelial Cells

**Figure 1:**
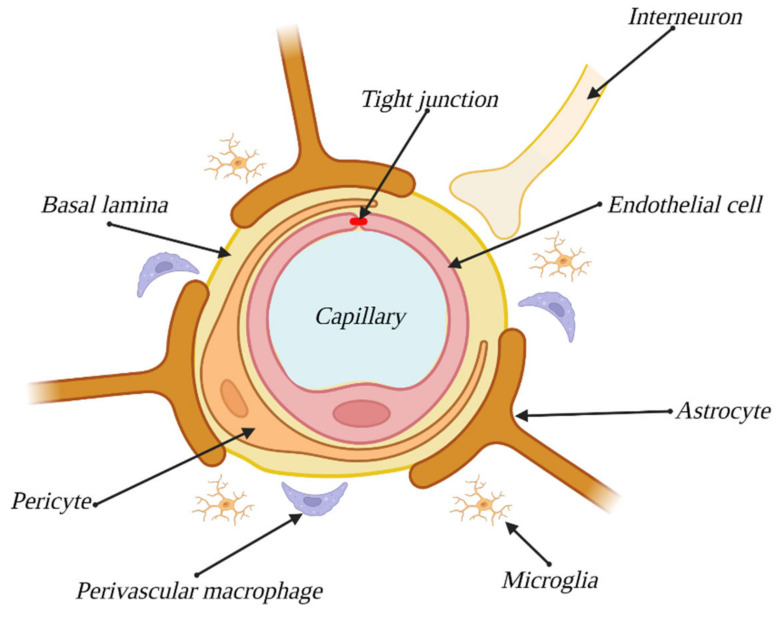
Blood–brain barrier components and composition. Created with BioRender.com (accessed on 22 February 2023).

ECs, which form the vasculature, should be considered the main component of the BBB. Although both the origins and overall structure of the endothelium of the BBB are the same as in that building the vasculature in the rest of an organism, the previous tend to express a series of qualities, specific only to them, such as TJs, high numbers of mitochondria, lack of fenestration or a low level of pinocytosis [7]. ECs along with the remaining components of the BBB are illustrated in Figure 1.

Moreover, the ECs of the BBB show a greater ability to express certain proteins responsible for forming tight connections between cells. These connections provide for the integrity of the BBB, and their presence and status are highly correlated with the levels of permeability presented by this structure. The connection region can be further divided into three subregions: the TJs region, adherens junction region and desmosome region [8], with each subregion characterized by a specific set of building proteins.

### 3.1. Tight Junctions

Crucial for the functioning of the BBB are the junctions located closest to the lumen of capillaries—the tight junctions, the presence of which makes for one of the most important parts of the BBB phenotype [9]. They create a hermetic barrier preventing various substances from passing through the EC layer via spaces between the cells, and they also separate the basal and luminal sides of ECs, leading to compartmentalization, creating what can be called the “blood side” and the “brain side” [10].

Components forming this junction can be divided into transmembrane proteins and cytoplasmic plaque proteins. The former are responsible for creating the Velcro-type connections (also known as zipper-type junctions), along with corresponding proteins from adjacent cells. The role of cytoplasmatic plaque proteins is to anchor transmembrane proteins to the cell cytoskeleton [8].

The transmembrane part consists of occludin [11,12], junctional adhesion molecules (JAM) [13] and representatives of the claudin family, while the cytoplasmatic plaque is built of proteins with the PDZ domain—ZO-1, ZO-2 and other proteins, e.g., cingulin [10].

### 3.2. Adherens Junctions

The second region of intercellular connections, located below the region of TJs, is the zone of adhesive junctions, responsible for contact-base inhibition of cell growth, polarization of the cell layer, and to a certain degree, maintenance of BBB permeability [9]. It has also been proven that proper functioning of these junctions is required for the formation of TJs [14]. As before, the structure of this connection can be divided into transmembrane proteins (mostly cadherins and nectin) and cytoplasmic plate proteins (catenins alpha, beta and gamma) [8]. Notably, adherens junctions and TJs are most likely structurally connected, assuming a determined interaction between alfa-catenin and ZO-1 [15].

### 3.3. Desmosomes

**Figure 2:**
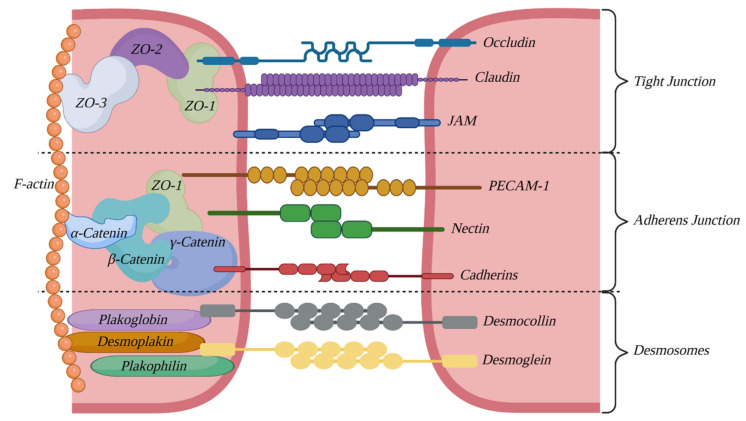
Junctional region between endothelial cells. Created with BioRender.com (accessed on 22 February 2023).

Last but not least is the desmosome zone of connections. In the context of the BBB, it is by far the least studied junction region; however, it undeniably contributes to the inhibition of BBB penetration, and at the same time, it carries out signaling functions, providing contact between adjacent cells [16]. As before, its structure can be divided into transmembrane proteins (desmocollin and desmoglein—cadherin family) and cytoplasmic plaques: plakoglobin, plakophilin and desmoplakin [8]. The connection region is illustrated on Figure 2.

### 3.4. Astrocytes

Astrocytes make for another cellular component of the BBB. These cells envelope the capillaries of the brain, remaining in contact with ECs via the basement membrane wherever the endothelium is not coated with pericytes. Thanks to their star-shaped, strongly branched morphology, they enable communication between ECs and neurons, where one astrocyte can remain in contact with up to 140,000 synapses [17]. In contact spots, they manifest a structure called end-feet, with the purpose of enlarging the area of contact [7]. Astrocytes play essential roles both during the differentiation of the endothelium toward the BBB phenotype and in BBB functional maintenance. They take part in all of the aforementioned aspects of barrier functions; they improve the integrity of TJs [18], express transport proteins (e.g., glycoprotein-P) [19] and increase the expression levels of barrier enzymes [20,21]. Astrocytes have been observed to influence the creation of capillary-like structures by ECs and pericytes [22], which proves that establishing the BBB phenotype demands the presence of these cells. Moreover, the interaction between ECs and astrocytes is bidirectional, and the endothelium induces the growth and differentiation of astrocytes [23], whereas in cocultures of those two cell types, increased levels of antioxidant enzyme expression, both in ECs and astrocytes, have been observed [24].

### 3.5. Pericytes

Pericytes, another cellular component of the BBB, are small cells enveloping the brain’s capillaries, covering 22–32% of the vasculature surface in this area [9]. The origin of pericytes is heterogenous and tissue-dependent, but most commonly described is their origin from mesenchymal stem cells. However, it has also been proven that brain pericytes originate from neural crests, indicating their ectodermal descent [25]. They are anchored in the basement membrane, remaining in contact with both ECs (through gap junctions) and other pericytes (via connections known as peg and socket junctions) [26]. They play a crucial role in maintaining BBB functions, influencing barrier permeability through the synthesis of TGF-β1 (transforming growth factor), VEGF (vascular endothelial growth factor), bFGF (basic fibroblast growth factor) and angiopoietin-1 [27]. The proteins listed above contribute to the creation of TJs through the synthesis of their components, claudin-12, JAM, ZO-1, ZO-2 and occludin [28]. They are also crucial for the expression of transporter proteins associated with the barrier function of the BBB, such as ABC G2, P-glycoprotein, multidrug resistance-associated protein, glucose transporters, L-amino acid transporter-1 and chloroquine resistance transporter [29]. Moreover, they take part in constructing the basement membrane, synthesizing a few of its components: collagen IV, laminin and glycosaminoglycanes [30]. Pericytes are also responsible for modulating the capillary blood flow by regulating the diameter of capillaries. This is possible due to expression of contractile proteins that build up pericyte cytoskeleton, i.e., filamentous actin (F-actin) and alpha smooth muscle actin (α-SMA), allowing them to constrict and relax in response to neuronal signals [31,32]. The role of pericytes is also to induce astrocyte polarization in the end-feet zone [26]. It is worth mentioning that pericytes exhibit properties of stem cells, and when treated with bFGF, they express markers characteristic of microglia and neuronal cells [33]. Pericytes have also been proven to differentiate into neuronal and endothelial cells after an ischemic stroke, proving their crucial role in the case of CNS injuries, as they are able to promote both neurogenesis and angiogenesis [34].

### 3.6. Basement Membrane

The last component of the BBB is the extracellular matrix (ECM), which forms the basement membrane (BM). Unlike the components described above, it is a noncellular structure constructed of proteins expressed by ECs and pericytes, mostly elastin, laminin, fibronectin and type IV collagen [35]. The BM envelopes ECs, separating them from pericytes and end-feet of astrocytes and pericytes and astrocytes from each other [36]. The BM participates in the maintenance of the BBB mostly as a structural base for remaining components and allows them to anchor thanks to adhesion receptors, which are characteristic of the basement membrane [37]. Malfunction of the BM leads to corruption of the EC cytoskeleton, which translates to impairment of TJ function and thus an increase in BBB permeability [38]. The chemical composition of the BM is regionally dependent (different compositions in various locations of the central nervous system) [25].

### 3.7. Neurovascular Unit

The components described above are considered direct parts of the BBB, but it is important to note the indirect influence of other kinds of cells, such as neurons, microglial cells and perivascular macrophages. These cells, along with previously mentioned BBB components, make up the neurovascular unit (NVU), the system responsible for maintaining the proper internal environment of the CNS through control and modulation of BBB functions and cerebral blood flow [25]. This system provides barrier elasticity, enabling meeting the CNS’s variable demand for nutrition and other substances. It is also partially responsible for proper formation of the BBB and maintenance of its functions [36]. The main mechanism initiated by the NVU is neurovascular coupling, also known as functional congestion, and a local increase in cerebral blood flow to satisfy the demands of the CNS.

## 4. Other Components of the NVU

As mentioned before, neurons, microglial cells and perivascular macrophages influence the functioning of the NVU to some degree; however, their importance is far less, and thus, they are collectively described below.

### 4.1. Neurons

Neurons are vital mostly in the induction of neurovascular coupling due to their high sensitivity to slight changes in oxygen and nutrient concentrations, as well as their ability to generate appropriate signals in response [36]. They have also been proven to influence EC differentiation toward the BBB phenotype [39,40]. After formation of the BBB, neurons regulate the expression and activity of efflux transporters as well as the circadian clock genes of the ECs, which subsequently regulate transport via the BBB. Neurons have been proven to regulate many other genes of the ECs, among them genes associated with transport, metabolism and focal adhesion [41].

### 4.2. Microglial Cells

In their native form, microglial cells are small, strongly branched, and they inhabit regions of the CNS [25]. In pathological states of organisms, they transform their morphology toward one of two possible phenotypes (M1 and M2), similar in terms of amoeba shape but differing in the spectrum of produced proteins. Phenotype M1 (proinflammatory) expresses proteins that increase the permeability of the BBB, which enables the penetration of leucocytes into the CNS, while phenotype M2 (anti-inflammatory) produces agents responsible for immunosuppression and angiogenesis [42]. Both phenotypes exhibit phagocytotic properties [43].

### 4.3. Perivascular Macrophages

Similar to microglial cells, macrophages are immunologic cells of the CNS that defend against pathogens [44]. They stay in contact with the remaining cells of the NVU, which most likely contributes to its proper functioning [25]. Similar to microglial cells, macrophages exhibit phagocytotic properties. Their ability to remove β-amyloid (a substance associated with Alzheimer’s disease) has been proven [45].

## 5. Transport through the BBB

**Figure 3:**
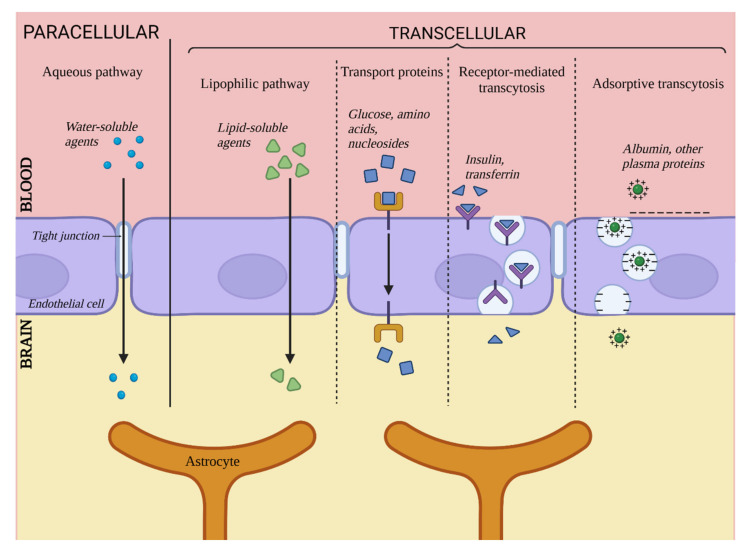
Transport via the blood–brain barrier. Created with BioRender.com (accessed on 22 February 2023).

The BBB is responsible for maintaining proper conditions in the integral environment of the CNS, delivering nutrients and oxygen as well as removing metabolites and carbon dioxide. In addition to substances required for proper CNS functioning, other chemical compounds pass through the BBB as well, e.g., barbiturates and ethanol [1]. This section is dedicated to discussing five main paths of particle transport through the BBB, illustrated in Figure 3.

First, transport through the BBB can be divided into paracellular and transcellular transport. Paracellular transport relates to particles passing through spaces between two adjacent ECs, and transcellular transport is a collective term for four pathways of transport directly via ECs.

### 5.1. Paracellular Transport

Unlike in ECs located in other parts of organisms, this particular pathway plays a relatively insignificant role in the context of whole transport through cell layers of the brain vasculature due to the presence of TJs. In physiological conditions, this pathway is limited to small hydrophilic particles and small ions. It has been determined that the maximum size of particles capable of passing through this pathway is approximately 10 Å [6]. Despite the limited role of paracellular transport in whole transport through the BBB, it is not to be overlooked during the search for potential carrying agents for substances targeted for the CNS. Since the low significance of this pathway is caused by the integrity of the TJs, it can become more prominent and available for larger particles in cases of pathological or artificially-induced integrity decrease [15].

### 5.2. Transcellular Transport

Transcellular transport includes four pathways leading directly through the EC body and is the primary means of transport through the BBB. It includes passive diffusion, transport via carrier proteins, receptor-related transcytosis and adsorption-related transcytosis [46].

#### 5.2.1. Passive Diffusion

This mechanism is responsible for the transport of lipophilic and amphiphilic substances by diffusion through the cell body. A substance’s ability to penetrate the BBB through passive diffusion is directly proportional to its lipophilicity [47]. This ability also depends on factors such as particle size, electric charge or ability to create hydrogen bonds, but lipophilicity remains the most important determinant [46]. This pathway is somewhat limited by the presence of ABC transporters, for which many lipophilic substances are substrates [47]. Examples of such transporters are P-glycoproteins, which are associated with the mechanism of multidrug resistance. They are located on the surface of ECs on their luminal side, and they capture substances trying to pass through the BBB, preventing them from achieving high concentrations in the CNS [19].

#### 5.2.2. Transport via Carrier Proteins

This pathway is responsible for transporting substances that exhibit a polar character and that are unable to diffuse via the cell membrane of ECs. Among other things, it takes care of components vital for the maintenance of proper CNS functioning, such as glucose, amino acids or nucleosides [47]. This takes place in the presence of carrier proteins secreted by ECs, many of which are highly specific for their designated carried substrate, e.g., GLUT1 (key carrier of glucose) [48]. Many others are less specific, recognizing certain functional groups of substances, e.g., carriers for LNAA (Large Neutral Amino Acids), which binds substances with carboxyl or α-amino groups [49]. Carrier proteins are members of two large protein families, solute carriers and ABCs [50]. It is believed that this pathway is most promising as a mean of targeted CNS therapy [46,47].

#### 5.2.3. Receptor-Mediated Transcytosis

This pathway is responsible for the transport of particles such as large peptides and proteins (e.g., insulin [51] or transferrin [47]). It is associated with surface receptors of ECs that bind specific ligands. Binding leads to the emergence of a cavity in the cell membrane and the formation of transport vesicles. This pathway is also related to the barrier function of the BBB since it has been reported that once the vesicle enters the cell’s body, it can fuse with a lysosome, which leads to degradation of the contained substance [52]; thus, it can be considered a neutralization mechanism for substances undesirable for the CNS. This pathway can also be utilized for delivering pharmaceutics into the CNS under conditions of designing vectors specific for EC receptors and binding said pharmaceutic to them [51].

#### 5.2.4. Adsorption-Related Transport

The fourth transport pathway described here is responsible for the transport of blood-derived proteins, e.g., albumin, that can undergo cationization - emergence of a positive electric charge that allows for interaction with the negatively charged cell membrane of ECs [53]. Next, similar to the previous path, the formation of a transport vesicle occurs, and the substance is transported inside the cell body. This pathway is less important for whole BBB transport since most cationized proteins are captured by the liver and excreted by the kidneys [47]. Nonetheless, it gives some hope for being used in potential CNS therapy by binding carried substances to a CPP marker (Cell Penetrating Protein). CPP markers possess a strong positive charge that activates transcytosis [54].

## 6. Criteria for Quality Evaluation of *In Vitro* Models

Once the structure and functioning of the BBB have been discussed, to further consider its models, certain criteria of its quality must be defined. Such criteria, based on the BBB’s characteristics *in vivo*, allow for determining the degree of correspondence between the discussed model and *in vivo* conditions. Aside from evaluating the quality and usability of existing models, formulating the criteria also allows, to some degree, predicting the further evolution of this branch of experimental methods. This section is devoted to discussing the qualities of the BBB phenotype, which are the same qualities that should be expected of a relevant *in vitro* model. Evaluation methods for these qualities shall be discussed as well.

One of the most important characteristics that should be expected of a reliable BBB model are its integrity and impermeability against substances undesirable in the CNS territory. On the other hand, to properly reflect the BBB’s properties, the *in vitro* model should be permeable to certain substances, similar to its *in vivo* counterpart. In other words, a reliable *in vitro* model must exhibit features of both barrier and transport functions of the real BBB. As stated, barrier function is implemented not only due to EC integrity but also through the secretion of specific carrier proteins and barrier enzymes. Therefore, their expression can serve as an evaluation method for models.

### 6.1. TEER Measurement

**Figure 4:**
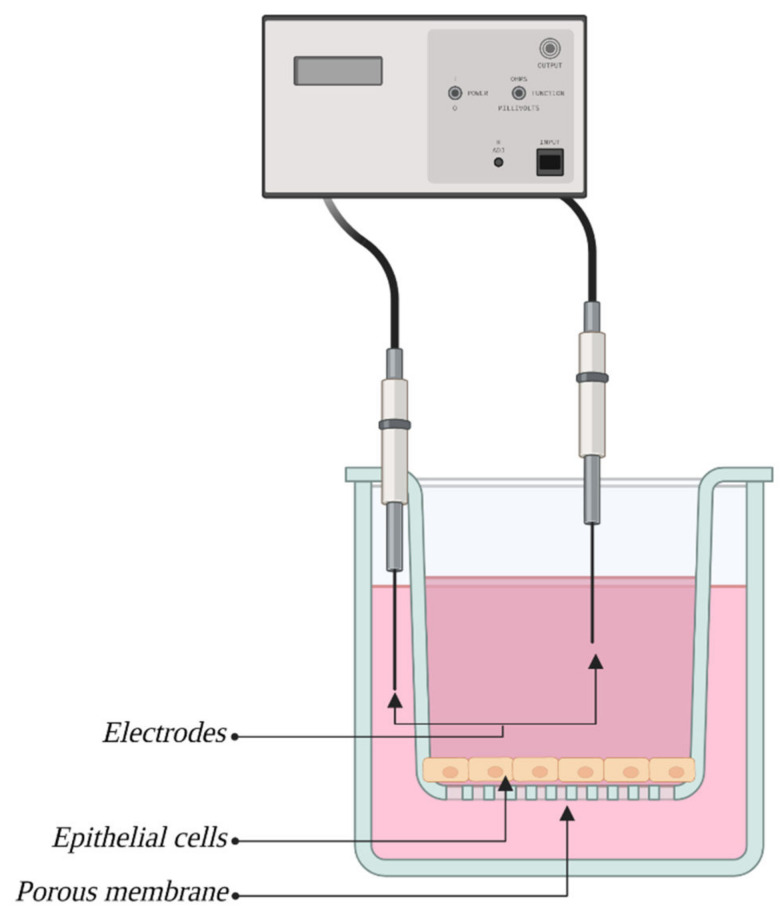
Transendothelial electrical resistance (TEER) measurement. Created with BioRender.com (accessed on 22 February 2023).

The first and most popular method of measuring membrane integrity, and thus permeability, is the measure of transendothelial electrical resistance (TEER). This method allows access to ionic conductivity via the paracellular pathway and is a great indicator of barrier integrity for the emergence of electrical resistance set by ECs derived from the existence of TJs between adjacent cells, and its value increases in direct proportion with the level of integrity of TJs. The most popular version of this method, the so-called “classic method”, consists of placing two electrodes inside of a culture container, one for each side of the culture insert with an EC membrane, as shown in Figure 4. Next, electrical current is applied and flows between the two electrodes, and the difference between the amperage on both of them is used to determine the electrical resistance of the EC layer. The resistance of the insert’s membrane and medium are also accounted for [55]. A popular modification of this method is ECIS (Electric Cell-substrate Impedance Sensing), where ECs are cultivated directly on electrodes [56].

The value of this measurement is expressed as Ωxcm^2^, since the EC layer area is of great importance for this measurement. The advantages of this method are its simplicity, low time consumption, repeatability and noninvasiveness [6]. The EC layer does not undergo destruction or any permanent changes, which allows it to be reused later. A wide variety of commercially produced systems for measurement simplifies studies even more and increases the repeatability of outcomes. Another advantage of this method is the possibility of employing it in dynamic models to obtain measurements in real time.

This method is fraught with some limitations, the major one being that the TEER depends strongly on the position of the electrodes, which does not allow to measure reliable values. Moreover, many limitations result from the following factors that can impair measurement values. First, values acquired from cocultures can be both overstated (with pericytes) and understated (with astrocytes) due to the presence of other types of cells [15]. Another limiting factor can be temperature. The optimal temperature for TEER measurement was determined to be 37 °C [57], and slight changes from this value during the measurement can result in impaired outcomes. To counter this problem, a mathematical formula has been created that allows for acquiring values independently of temperature fluctuations [58]. Values can also be impaired by lack of shear stress (which will be discussed thoroughly in the section dedicated to dynamic models). Following possible sources of impairment in culture passages, which gradually decrease acquired values [59], the medium composition, especially additives such as ions or cAMP [60], and the composition of the culture membrane can determine the BBB phenotype, similar to the composition of ECM *in vivo* [6]. Additionally, the longer since the founding of the culture, the higher the acquired values are [61].

The developed models exhibit high volatility of TEER values, which can depend on previously described factors and stresses the need for a maximally unified method of measurement for pharmaceutical reasons. Acquired values are also usually significantly lower than those from *in vivo* models; for the human barrier *in vivo*, the approximate values have been estimated at 5000 Ω × cm^2^ [62], while those observed in most *in vitro* models oscillate between approximately 100–2000 Ω × cm^2^ [15].

To overcome these limitations, there are commercially available TEER measurement systems, such as Epithelial Voltohmmeter (EVOM), which avoid the charging effects on the electrodes and the cell layer. Nevertheless, the system highly depends on the electrode positions and requires very careful handling of the electrodes [63].

Impedance spectroscopy is another approach which gives a more accurate representation of TEER values than traditional systems due to reducing the impact of electrode-medium interfacial resistance on impedance calculations [55].

### 6.2. Transmittance Coefficient

Another popular method of integrity and permeability evaluation for BBB models is calculating the transmittance coefficient (P). This measurement relies on the use of marker substances with known molecular masses that are placed in a culture container with a membrane in its top section, representing the luminal side of capillaries. Next, the concentration of these markers is assessed on the other side of the membrane on the “abdominal” side. The acquired value is then divided by the original concentration of the substance, area of the EC layer and time of incubation, resulting in the coefficient P. Widely used marker substances are FITC-dextran and, even more, sodium fluorescein. For fluorescein, values of coefficient P of approximately 10–6 cm^2^/s are considered satisfactory [9]. Unlike the TEER measurement method, which measures the ionic conductivity of the paracellular pathway, the transmittance coefficient defines the layer’s permeability for water and the size of its pores [15].

### 6.3. Detection of Tight Junctions

As mentioned before, the presence of TJs is one of the primary parameters of the BBB phenotype, playing a key role in maintaining barrier function. The two following methods of *in vitro* model evaluation are based on the detection of these junctions. The first one employs the freeze fracture technique and electron microscopy. The studied material needs to be prepared by rapid freezing followed by creating cracks to expose structures of interest. Thanks to this technique, it is possible to observe the alignment of the cell structure on a flat surface [64]. In terms of *in vitro* BBB models, the object of interest should be transmembrane filaments present on the surface of ECs [65]. This method allows for precise localization and confirmation of the presence of TJs between ECs; however, compared to TEER measurement, the disadvantage is that once prepared, cells cannot be further used. Additionally, this technique cannot be used for real-time control.

The presence of TJs can also be detected with immunofluorescence staining, which allows visualization of the marker proteins of junctions, such as occludin, claudin family representatives or ZO-1, which are then observed with confocal fluorescence microscopy. The visualization of proteins relies on binding them with specific antibodies marked with fluorochromes [66]. The limitations, similar to the case described above, are the inability to conduct real-time culture control and to further use stained cells [15].

### 6.4. Expression of Carrier Proteins

Many carrier proteins constitute important elements of the BBB phenotype, contributing to maintaining both barrier and transport functions. In a similar manner, the *in vitro* models should also be able to express those proteins, especially members of the two aforementioned families, ABC and solute carriers [50].

### 6.5. Barrier Enzyme Synthesis

Another determinant of the BBB phenotype is the production of barrier enzymes by the ECs responsible for the deactivation of toxic substances. Similarly, *in vitro* models should present the expression of those enzymes, such as alkaline phosphatase (ALP), γ-glutamyl transpeptidase [67], acetylcholinesterase and monoamine oxidases [9].

### 6.6. Practical Requirements

Aside from the requirements of a theoretical nature mentioned above, resulting from following the goal of imitating *in vivo* conditions as precisely as possible, other requirements that are vital for selecting a proper research model for conducted experiments must be considered.

One of the most desired characteristics of the *in vitro* model is meeting the high-throughput screening criteria (HTS). This refers to the ability to conduct a high number of tests simultaneously, employing robotic methods and software capable of processing and controlling large amounts of data. Meeting the HTS criteria is required for a model to be utilized in large-scale pharmaceutical testing.

Another desirable property of the *in vitro* model is its ability to evaluate the parameters of the generated model, as mentioned before in this chapter, or even visual control of changes occurring inside the utilized research system. Additionally, aside from controlling the effects of different factors affecting studied cells, the system should allow for manipulating the conditions of the internal environment of the model, the chemical composition of the medium, or other blood substitute, and values of shear stress.

Further characteristics of models that require consideration are its cost, complexity (which sets a certain level of abilities required from the research team), and the time needed for establishing the model as well as its lifespan—meaning how long it will maintain the vitality of cells and the BBB’s phenotype.

In summary, *in vitro* models should reflect two primary functions of the BBB, both the barrier and transport functions. To cope with those expectations, the model should express a wide variety of proteins, such as those building TJs, adherens junctions and junctional adhesion molecules, carrier proteins, pumps, surface adhesion proteins, chemoreceptors, mechanoreceptors and enzymatic barrier proteins. The permeability of the barrier should correspond with values observed *in vivo* in terms of each transport pathway. Models should also allow for realistic interactions between components of the NVU, as well as their exposure to shear stress, immunologic cells, growth factors and other factors inducing emergence of the BBB’s phenotype. It should allow for research of both physiological and pathological conditions, whereas if it is for pharmaceutical and medical tests more than strictly scientific research, it should meet the HTS criteria, be relatively cheap, simple to establish and long-lasting. It is important that the model provides repeatable results and maximally enables control over culture environment parameters. It should be noted that not all models need to express the entirety of characteristics that could be implemented. It is thus crucial to assess whether a certain characteristic is vital, desirable or perhaps negligible in order to avoid redundant complexity or expenses.

It is important to note a kind of schism in the direction of development of BBB *in vitro* models. Two courses can be pursued by researchers, and along with technological progress being made, these two courses will drift further apart. On the one hand, the interest in a profound understanding of the BBB’s mechanisms of functioning is obvious, and models designed to imitate the BBB more precisely are gradually becoming more complicated and complex. Development of these models leads to implementation of subsequent cellular and noncellular components that contribute to the formation of the BBB phenotype. The limitations of this approach are the high cost of the designed models, the high skill required for establishing and maintaining them and the low level of repeatability and ability to compare results between independent laboratories.

On the other hand are models created for pharmaceutical research. Creating new drugs and therapies targeting the CNS demands the testing of large numbers of new compounds. Models designed in accordance with this approach are ultimately cheap, easy to use and recreate, and the results achieved thanks to them are reliable and repeatable, in other words, compliant with HTS requirements [6,68,69].

## 7. Techniques of *In Vitro* Modelling

The following section focuses on discussing *in vitro* models, the development of techniques related to them and evaluation of their utility for different research approaches. It should be noted that, at this moment, a perfect model does not exist; thus, only knowledge of the advantages and disadvantages of different models provides an opportunity to select the proper model for planned studies, research team skills and experience or equipment and financial limitations.

### 7.1. Artificial Membrane Models

The two most popular methods in this context are Immobilized Artificial Membrane (IAM) [70] and Parallel Artificial Membrane Permeability Assay (PAMPA) [71]. Their purpose is to imitate the characteristics of the cell membrane to study the permeation of substances through it. They rely on artificial membranes by covalently binding phospholipids characteristic of biological membranes to highly porous films. Naturally, such models are only capable of visualizing passive transport [72]; thus, they cannot be used to analyze carrier-related transport or barrier properties resulting from enzyme expression. Nonetheless, these systems can play an important role during early stages of pharmaceutical research because they are relatively inexpensive, easy to establish, repeatable and meet the HTS criteria [73].

Another technique that does not rely on cell culture is utilizing isolated membrane vesicles. In *in vivo* conditions, these vesicles take part in different pathways of BBB penetration, both into the CNS and out of it. Because of this, vesicles are isolated from either the luminal or abluminal sides of capillaries to study their role in the transport of substances such as glucose, amino acids, electrolytes [74] and other substances related to ABC transporters [75]. The primary disadvantage of this technique is its high cost resulting from the enormous demand for biological material, since fragments of cells that form vesicles are most commonly derived from bovine brain tissues due to their size. To gather the material required for a standard series of transport-related tests, approximately 100 brains [74] are needed at once, which, apart from high costs, also raises doubts of an ethical nature. Even so, this model makes for an honest method of analyzing vesicle-related transport, asymmetric distribution of functional carriers at both sides of the EC layer or changes that transported substances undergo inside the vesicles.

### 7.2. BBB Models Based on Cell Cultures

In *in vitro* models established thus far, cells of different origins are utilized. Mostly in use are brain capillary-derived ECs, both human and animal, but the alternative can be ECs of different origin, e.g., umbilical cord ECs as well as nonendothelial, e.g., MDCK cell lines (Madin–Darby Canine Kidney) or Caco-2, derived from human intestine cells [68]. In some models, stem cells are employed, with their differentiation induced toward ECs [76].

Epithelial-originating cells, such as MDCK or Caco-2 cells, express some functional similarities to cells building the BBB, such as the constituting of TJs and carrier protein synthesis; however, both the junctions and the proteins show structural differences compared to ECs of brain capillaries [77]. Even so, the level of permeation of substances achieved by these models shows a strong correlation with values reached with ECs [78,79] in terms of paracellular transport, passive transcellular diffusion and active transport, e.g., glycoprotein-P-related transport. The TEER values reached by these models, comparable to *in vivo* conditions, show that this approach can be implemented in BBB research [80]. Advantages in this case are relative ease of establishing a model, based on many developed protocols, low costs and high repeatability of the results thanks to the usage of cell lines. These models prove inadequate in terms of studying the interaction between components of the BBB or NVU or the influence of physiological and mechanical factors on BBB cells. In summary, models relying on nonendothelial cells can serve as alternatives for ECs in research on BBB transport, especially its early stages, and they need to be applied carefully, with their limitations regarding EC imitation kept in mind.

Another alternative for brain ECs is the employment of ECs derived from the human umbilical cord—HUVECs (human umbilical vein endothelial cells) [9]. The advantage of these cells is mainly their human and endothelial origin. They establish TJs, although through comparison with brain ECs, their limited ability to visualize the BBB becomes apparent. Moreover, they express lower values of TEER and higher permeability, and they do not express the genes responsible for immunoregulation or angiogenesis or synthesize growth factors (appropriate for brain ECs) [81]. Despite these limitations, they have proved useful in certain studies [82].

Another approach is to produce ECs with the correct properties by employing pluripotent stem cells. This solution allows bypassing the problem of the limited availability of human primary brain cells. Stem cells are induced to differentiate toward ECs using a variety of methods, e.g., through coculturing with the cell line OP9 (mouse bone marrow) [83]; however, to give them additional characteristics of the brain endothelium, a culturing method has been proposed that assumes the creating of an environment imitating the brain of an embryo, followed by immunochemical selection of cells showing the BBB phenotype. Cells acquired this way present many markers typical for the BBB, interact with astrocytes and show qualities of barrier and transport functions on levels close to those achieved with human primary cells [84]. It is an interesting approach, a response to the increasing need for human models while steering clear of availability limitations of primary cells and a vast majority of quality issues of cells lines (discussed later). The main disadvantage of this method is the need to differentiate cultures, which increases the level of required skills and overall cost of the experiment.

Aside from choosing cells for an experiment, an important matter is also the chemical composition of both the culture medium and porous membrane on which cells are cultured. The function of the membrane in culture is more closely discussed in part by focusing on the static model; however, the magnitude of this aspect should be noted at this point. Thus far, the composition of both the medium and the membrane have been determined by technicalities, such as the correct pore size, membrane thickness or ability to nourish the cells [6]. However, those two components of *in vitro* models are de facto supposed to imitate the extracellular matrix and basement membrane that accompany the cells *in vivo*. As mentioned before, these elements influence differentiation toward the BBB and maintain its functions, while their chemical composition is regionally dependent. Thus, precise analysis of these structures is needed to most accurately reflect their *in vivo* counterparts.

Independent of their origin, cells utilized for constituting models can be divided into two groups: primary cells, i.e., cells isolated from their native location in an organism, and cell lines, i.e., cells that were immortalized, usually by infection with lentiviral vector containing big-T antigen or via transfection with adenoviral gene E1A [85]. Both solutions carry possibilities and limitations, and both find their uses in different kinds of studies. It should be noted that this distinction refers to brain ECs, most desired for BBB research; all EC surrogates mentioned before, and every other cell type of the NVU, such as astrocytes, pericytes or neurons, utilized for establishing coculture models, are discussed later in this part.

#### 7.2.1. Primary Cells

Primary cells are utilized in *in vitro* models because they present high values of TEER, low permeability and expression of carrier and barrier proteins at levels corresponding to the *in vivo* situation [32,85,86]. Primary ECs originate from isolated brain capillaries and can be used to establish a culture of cells required for a BBB model. However, many studies, mainly related to the biochemical and biomechanical mechanisms of the barrier, have been conducted directly on isolated capillaries cultivated *in vitro*. Isolated capillaries contain ECs enveloped with basement membranes along with pericytes and astrocytic end-feet. They are cleansed in a process consisting of homogenization, enzyme treatment and column filtration [87]. Due to the presence of many BBB qualities, on levels compared to *in vivo* conditions, they have been utilized in many studies focused on BBB functions, such as the operation of xenobiotic-removing pumps [88], protein phosphorylation induction [89] or transporter activity of glycoprotein-P [90], and both human- and animal-originating capillaries have been used. Another advantage of isolated capillaries and primary cultures is their possible use for establishing models of diseases, exposing qualities characteristic of pathological states such as brain cancer [87,91].

Even though the cells maintain their metabolic properties, the isolation process and subsequent temporal metabolic shortages can negatively affect barrier integrity. It should be noted that a high complexity level of the isolation process and cleansing requires a properly qualified research team. The procedure itself is burdened with high contamination risk [68]. Another disadvantage is the relatively short time and low number of passages, after which ECs in capillaries and primary cultures lose their barrier properties [69], and a slow tempo of proliferation in primary cultures is observed [6]. The availability of study material poses a problem as well. Animal-originated material is quite easily obtained, but its acquisition leads to excessive use of animals and moral dilemmas; additionally, it is less adequate for establishing models focused on researching human barriers. Human-originated cells are better suited for pharmaceutical tests, although they are not easy to access and usually come from sections or surgically removed tissues; in both cases, they are usually in a pathological state, which can disturb their function. Utilizing primary cells also leads to lower levels of repeatability between different laboratories since cells derived from different donors may differ. This approach is also relatively expensive because of the high level of required skills. These constraints lead to a lack of compliance with HTS requirements, thus limiting the use of these methods for large-scale pharmacological research.

#### 7.2.2. Cell Lines

An alternative for primary cells is utilizing immortalized cell lines derived from both human and animal donors. Their greatest advantage is the relative ease of usage and low costs of maintaining cultures. Immortalized cells of set lines can be cultured for a longer period of time with little to no decrease in viability parameters and expression of markers in comparison to primary cell cultures. However, the longevity level may differ from line to line; cells originating from lines are generally considered more suitable for long-lasting models.

Cell lines also provide the possibility of achieving highly repeatable results. Their main disadvantage is expressing, with few exceptions, much lower values of TEER and higher permeability than primary cells [69]. Currently, there are many lines of ECs derived from mice, rats, bovines and swine, and in 2006, the first stable and precisely characterized human line was described: hCMEC/D3 [86,92,93]. The three lines most commonly employed in *in vitro* models are RBE4 (rat-derived), derived from Sprague–Dawley rat ECs; bEnd.3, isolated from the endotheliomas of BALB/c mice [94]; and hCMEC/D3, as mentioned above. In particular, the human line is an object of research interest because of its great potential to mimic the human BBB. It does present relatively low values of TEER, but it also shows expression of many marker substances, characteristic for barrier phenotype, such as JAM, ZO-1, claudin-5 and other proteins [93]. Another approach is employing human pluripotent stem cells (hPSC)-derived ECs. These offer an answer to scarcity of primary cultures of human ECs. Nevertheless, they express markers specific for endothelium, and the process of inducing their endothelial phenotype is relatively complicated, making this application interesting yet still under development [32,86,95,96,97].

### 7.3. Static Models

Static models are the backbone of *in vitro* testing, and the simplest of them were constructed as this experimental method branch was born, evolving in time toward increasingly complex layouts. Early models were built upon a limited understanding of BBB functions and were meant to be versatile tools for researching this structure. Along with the development of neurobiological sciences, new models emerged that were more precise in mimicking the *in vivo* conditions and allowed for increasingly specialized research of specific phenomena occurring in the BBB [98].

**Figure 5:**
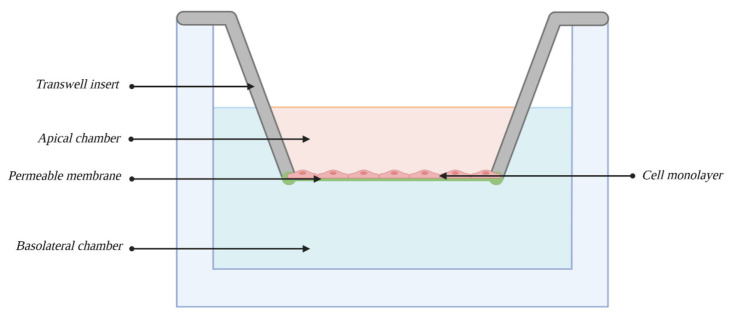
Transwell apparatus. Created with BioRender.com (accessed on 22 February 2023).

Static models, also called 2D models, are based on the employment of a Transwell insert system (see Figure 5), vessels or well plates consisting of two chambers divided by a porous, semipermeable membrane, usually made of PET (polyethylene terephthalate), on which the cells are cultured. The top chamber of this model usually represents the capillary lumen, while the bottom side represents the territory of the CNS. A semipermeable membrane allows bidirectional substance transport, and depending on the size of the pores, cell migration is also possible [69]. Thanks to the use of such a culture system, it is possible to measure the permeability of the EC layer toward certain substances, and more complex systems have integrated electrodes for TEER measurements. The greatest advantage of models employing Transwell inserts is their relatively low cost and potential compliance with HTS requirements. To a certain degree, they can be considered easy to establish and maintain; however, the more complex the model, the higher the skills needed [94]. Nonetheless, they are still simpler than the dynamic models discussed later. The biggest disadvantage of all static models is that they ignore the influence of many biomechanical and physiological factors on BBB development [68], mostly stress exerted on the EC layer by the blood circulating through brain capillaries.

#### 7.3.1. Monocultures

The first-generation static models, also referred to as monocultural models [6], are the simplest and relatively primitive way of imitating the BBB. ECs derived directly from tissues or cell lines are cultured on the top insert until they reach confluency. The main advantage of these cultures is how easily they are used for constructing a model and their low cost; however, these models present relatively low values of TEER and high permeability. The reason for this is mostly a lack of interactions with other BBB cells, mainly astrocytes and pericytes. This leads to restricted formation of TJs and irregular cell adhesion [68]. An additional issue for these models is the “edge effect”, which is a great impairment of the barrier qualities of the EC layer near its edges [85] because cells are not able to establish a TJ with culture vessel walls, leading to significant yet artificial overstatement of permeability. Because of the lack of factors produced by cells accompanying ECs in the BBB, which are responsible for both BBB development and maintenance, the ECs in these models lose their barrier properties relatively quickly [68].

However, in spite of numerous limitations, the models described above serve as a tool for barrier quality, permeability, TEER values and composition of the culture membrane. Considering the most important discoveries accomplished with those models, Lazarovici points to research into the influence of high concentrations of hydrocortisone and cAMP (cyclic adenosine-3′,5′-monophosphate) on TEER value increases and the usefulness of polycarbonates in semipermeable membrane production. Simultaneously, the same author mentions that the main conclusion based upon research on first-generation models was that they expose high permeability and low TEER value, ergo, low usefulness for large-scale pharmaceutical research [6]. This dictated the need to constitute more advanced models, employing other cellular components of the BBB, although it should be noted that monoculture models can still be seen as potential tools in screening tests, as it was proven that it is possible to create a monoculture model exhibiting satisfying levels of TEER values (800–1800 Ω×cm^2^) and the presence of some transport proteins [99,100].

#### 7.3.2. Coculture

Cocultures, also called the second generation of *in vitro* models of the BBB, were an attempt to implement the influence of other cellular components of the BBB, accompanying ECs *in vivo*, mainly astrocytes and, to a lesser degree, pericytes [69]. This group can be generally divided into contact and noncontact cultures [101]. For contact cultures, ECs are cultured on the top side of the semipermeable membrane, and astrocytes are seeded on the bottom side, which allows for direct contact, although the coverage of ECs by astrocytes represents just a fraction of the analogical situation *in vivo* because of the limited density of membrane pores. For the noncontact cultures, ECs remain on the top side of the membrane, and astrocytes are cultured at the bottom of the culture vessel. In this case, the interaction between cells is based on the permeation of astrocytic factors through the membrane. An alternative way of implementing the influence of astrocytes on ECs is to employ a culture medium conditioned by astrocytes containing regulating factors characteristic of astrocytes. However, as stated, the astrocyte–endothelium interaction is bidirectional, so the described approach eliminates the possibility of establishing a truly synergistic influence of both cell types on the emergence of the BBB phenotype.

Astrocytes used in these models can be derived either from primary culture or cell lines. Capabilities and limitations of both types are in many cases convergent with those described for ECs in terms of cell vitality, loss of some metabolic aspects or the ability to produce specific factors, but other complications can arise as well. For example, C_6_-glioma, a widely used astroglial cell line derived from rat glioma, presents a significant defect, similar to other astrocytoma-derived cells, in the production of VPF (vascular permeability factor), which contributes to a great decrease in the barrier properties of the BBB in coculture [6].

Research with coculture models with astrocytes has demonstrated the key role of astrocytes in TJ formation, decreasing membrane permeability and obtaining high TEER values [86,100,102,103]. They were also used for designating the minimum diameter of semipermeable membrane pores that allows for penetration of the membrane by astrocyte end-feet and direct contact with ECs (> 0.8 µm) [6]. However, those models still exhibit relatively high permeability, which limits their usefulness for pharmaceutical research, and they still make an important step on the path of *in vitro* modeling, representing the synergistic influence of different compounds of the BBB on its development and maintaining its functions.

#### 7.3.3. Multicultures

The most complex, build-wise, are models based on multicultures, also known as third-generation models. They address the important influence of NVU components on forming and maintaining the barrier phenotype of ECs, pericytes, to a higher degree than in models described above and, above all, neurons, with a particular focus on the synergic influence of astrocytes and pericytes [86,104,105]. These models exhibit the lowest permeability and the highest TEER values of all static models [101]. They can be conducted in different setups; ECs are cultured on the top side of the membrane, as before, astrocytes can be seeded on the bottom side and pericytes at the bottom of the vessel, or conversely. In some cases, astrocytes and pericytes are cultured together at the bottom side of the membrane. Neurons are usually cultured at the bottom of culture vessels, sometimes alongside other cell types [69].

The models described above have been used to research the synergistic influence of NVU components on establishing the BBB, the reasons for increased permeation [106], and the influence of neurons on the BBB [107] and have allowed for further research on pericytes. There is no doubt that among static models, this particular group offers the best reflection of the BBB, mainly in terms of multidirectional dependencies between its components. Their constraints are relatively complex structures leading to high costs and more difficulties with maintaining cultures, which can limit their usefulness in HTS tests.

In summary, static models are potentially appropriate models for HTS testing in pharmacological research; however, they each bring certain opportunities and limitations. On the one hand, the low costs and level of required skill for monoculture models speaks in favor of them under conditions in which satisfying levels of permeability are obtained. Even a negligible reflection of *in vivo* conditions caused by a lack of other cell types does not exclude this group, although highly specialized models for specific research goals should be constructed. It has been proven that, despite attaining significantly higher permeability levels than multicultures, monocultures are capable of exhibiting some aspects of transport function on a satisfying level, relatable to more complex models [108]. On the other hand, more advanced models offer interesting research capabilities thanks to more accurate reflection of the BBB and associated intercellular interactions; thus, these models are more likely to play a crucial role in further study on these issues. Their significance for pharmaceutical testing is, for the time being, constricted due to highly complicated culture maintenance and relatively high costs. It should also be noted that one of the great limitations of static models is the lack of biomechanical factors.

### 7.4. Dynamic Models

The answer to the rising demand for increasingly adequate BBB modeling is subsequent attempts to implement physiological and biomechanical factors into models known as dynamic. Such models are currently the most reliable way of imaging the BBB and an invaluable tool for studying its properties. Their advantage over static models comes from implementing previously overlooked factors that regulate BBB development, maintenance and function and are caused by blood flowing through capillaries, mainly by the emergence of shear stress. However, these models are hard to maintain due to their technological advancement, especially for laboratories that do not possess strictly biomechanical/bioengineering facilities, and high costs may also be a limitation. In spite of these difficulties, throughout recent years, many models based on these ideas were designed, and they will be discussed in the following chapter.

#### 7.4.1. Shear Stress

The influence of shear stress associated with blood flow on the emergence of a tight BBB is relatively well described [109]. Among others, it has been proven that shear stress leads to the formation of tighter TJs and adherens junctions, lowers barrier permeability toward albumin and macromolecules, and decreases the number of so-called leaky junctions by lowering the levels of apoptosis and proliferation in culture [110]. Shear stress also increases the level of secretion of various proteins associated with both barrier and transport functions, including multidrug resistance (MDR), transport proteins specific for glucose, monocarboxylic acids, integrins and surface adhesion proteins [111]. Shear stress has also been observed to influence endothelium morphology itself, i.a., inducing a larger cell size, flattened shape, higher number of microfilaments and emergence of a convex cell membrane called clatrin pits, associated with clatrin-dependent endocytosis [68]. The multifaceted influence of shear stress on ECs is possible thanks to their many mechanoreceptors, which process mechanical stimuli to biochemical signals, e.g., ionic channels and integrins. Such notable meaning of shear stress clearly shows why modern and reliable models of the BBB should be designed with this force taken into account.

#### 7.4.2. Dewey and Bussolari Model

The first attempt to implement the influence of shear stress on ECs was a conical apparatus designed by Dewey and Bussolari in 1982. This device allowed for rotation of the conical culture vessel, leading to the formation of centrifugal force and thus contact stress from medium pressure on the EC layer. The device made it possible to study cell responses to varying levels of stress, since values of shear stress could be regulated by adjusting the speed of rotation and angle of the cone. This contraption was mainly utilized to study the influence of shear stress on cell morphology, lifespan in culture, different times of EC exposure to stress and varying medium composition. As noted by creators of this device, it was not intended for this model to be a precise reflection of *in vivo* conditions; it did not implement other factors of the BBB’s environment, but nonetheless, it was an important step toward more complex models addressing physiological influences on BBB functioning [112].

#### 7.4.3. Parallel Plate Flow Chamber Model

**Figure 6:**
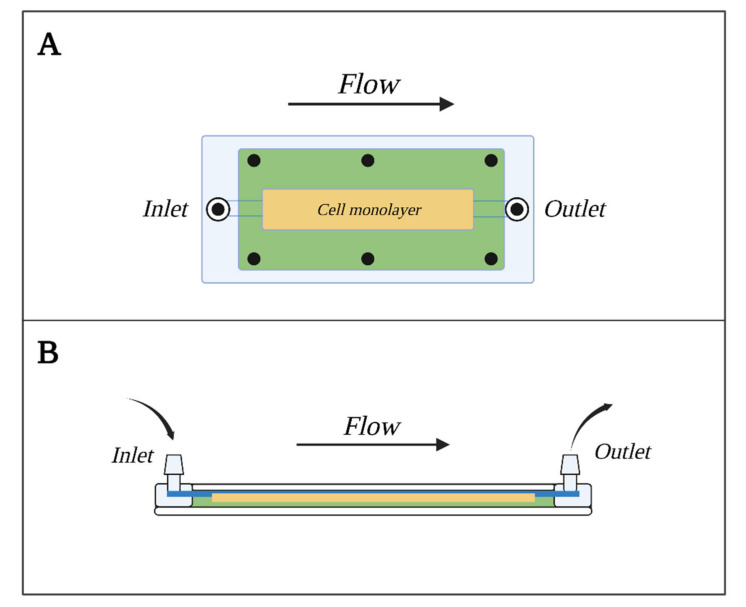
Parallel Plate Flow Chambers (PPFC) model. (**A**)—view from above, (**B**)—side view. Created with BioRender.com (accessed on 22 February 2023).

Another approach to BBB modeling with shear stress taken into account is the parallel plate flow chamber (PPFC) shown in Figure 6. This consists of two plates, out of which the bottom one, usually made of glass, is coated with adhesion factors (collagen, fibronectins, etc.) and serves as the sowing area for cells. The top plate, made out of transparent polycarbonate, allows for visual observation. Plates are divided with a silica gasket, the width of which determines the diameter of the model’s flow channel. The channel that carries the medium responsible for forming the shear stress runs from the entry valve to the exit valve, parallel to the ECs layer. More advanced PPFC models are equipped with a semipermeable membrane, similar to the one found in Transwell inserts, that divides the channel into luminal and abluminal sides [113]. Such models allow for precise designation and control of the intensity of flow and force applied to ECs as well as determination of flow type, laminar flow being the most desired [114].

Models from this group were mostly used to study adhesion, intercellular transport interactions between ECs and leucocytes, and the influence of shear stress on barrier abilities and morphology [68,114,115].

The advantages of this model are, among others, the ability to generate shear stress in a highly controllable and measurable manner, simple construction, low cost, relatively low number of cells required for establishing the model and the possibility of conducting visual control with a microscope as well as of employing immunochemical staining. The limitations are the negligible influence of other cellular components of the BBB and the inability to measure TEER.

#### 7.4.4. Dynamic *In Vitro* BBB Model

**Figure 7:**
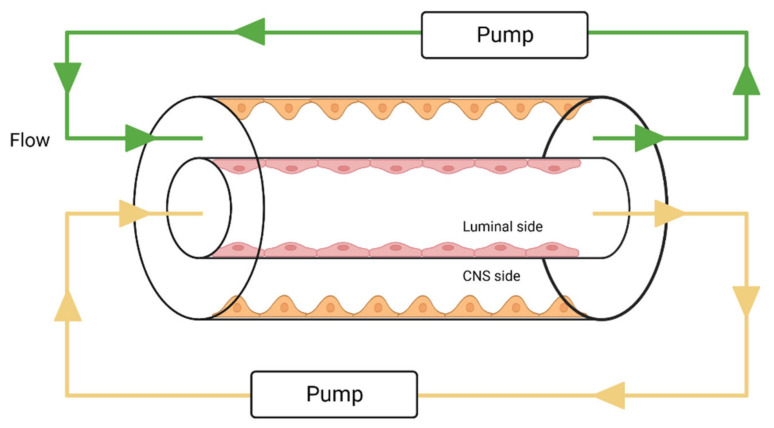
Dynamic *in vitro* BBB Model. Created with BioRender.com (accessed on 22 February 2023).

Just as with static models, the next step for dynamic models is to move from monocultures to cocultures to enable intercellular interactions to mimic the BBB more reliably. The first of such models is known as the Dynamic *in vitro* Blood–Brain Barrier (DIV-BBB) model, which is based on cultures inside a system of fibers, imitating brain capillaries, where ECs are cultured on the inside and astrocytes on the outside [116]. Through the lumen of fibers flows the medium, responsible for generating shear stress, pumped inside by pumps regulating the intensity of flow and causing medium pulsation. Additionally, the flow can be regulated by manipulating the diameter of the fibers and the medium’s viscosity [85,117]. The basic scheme for this type of model is shown in Figure 7.

Thanks to implementing both the shear stress influence and ECs–astrocyte interactions, these models achieve high TEER values, low permeability against many substances, and higher levels of expression for various transport and pump proteins. The already mentioned role of shear stress in regulating apoptosis and proliferation allows for the use of these models in long-term studies and also enables strict regulation of experimental conditions, which makes these models perfect research tools for studying the long-term influence of physiological and pathological values of stress in brain capillaries [118]. They have also been employed in research on inflammatory mechanisms [119] and the migration of cells [117].

Limitations of this model lead to its negligible usefulness in screening pharmaceutical tests because it does not comply with the HTS requirements, is relatively costly and complex, and the process of establishing this model is relatively time-consuming. Compact construction of a model precludes visual control over cells inside fibers and evaluation of cultured EC layer permeability in real time. To conduct morphological studies, the fiber needs to be irreversibly removed from the system. Further limitations are caused by how the fibers are built. First, they are commonly made of lipophilic materials, e.g., polypropylene, which can restrict the transport of many lipophilic substances. Second, the diameter of fibers is greater than *in vivo*, so the model seems more suited for studies on phenomena occurring in larger vessels. Finally, to establish this model, a high number of cells and large volumes of reagents are needed, thus generating costs. The complicated process of establishing and enabling the model also leads to restrictions in the repeatability of measurements, as it is difficult to achieve two identical models [68,111,118].

#### 7.4.5. 3D—Extracellular Matrix Model

**Figure 8:**
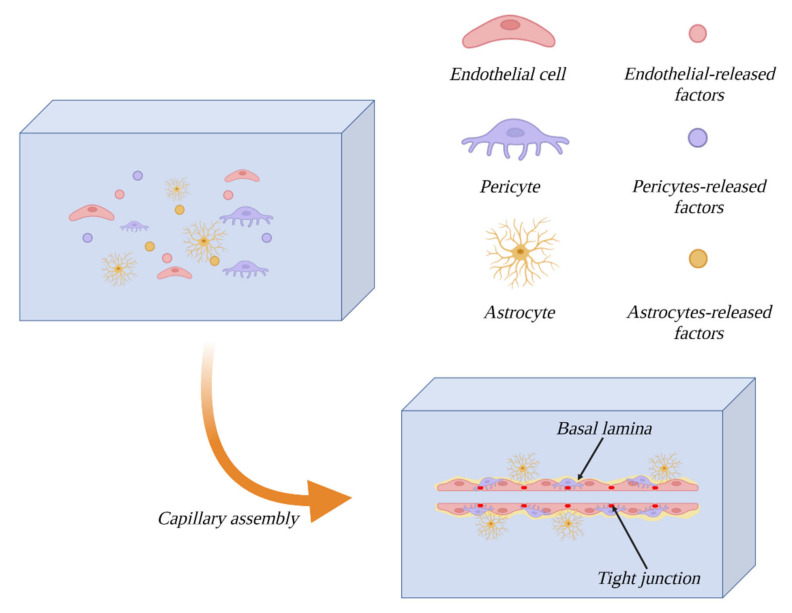
Three-dimensional extracellular matrix (3D–ECM) model. Created with BioRender.com (accessed on 22 February 2023).

Another approach to dynamic modeling of the BBB is to conduct spatial cultures in gels imitating the extracellular matrix (ECM). This approach is an answer to the aforementioned limitation of *in vitro* models caused by omitting the importance of the microenvironment for BBB cellular component development. The culture gel, in which the cells are suspended, consists mostly of proteins natively associated with the ECM, e.g., laminin, collagen, elastin, fibronectin, etc. [120,121]. The main advantage of this model is enabling the emergence of a biochemical gradient of trophic factors necessary for proliferation and intercellular communication [85]. Its limitations are mainly the complicated production process and high cost of use. It should be noted that it does not fully mimic *in vivo* conditions, and it is best suited for research on the influence of different medium components on angiogenesis, proliferation and cell migration, mainly cancerous cells [122]. The formation process of this model is illustrated in Figure 8.

An interesting variation of this model type is a model described by Campisi et al., which is a model of vasculogenesis in fibrin gel. The cellular components of this model are pericytes, astrocytes and human-induced pluripotent stem cell-derived ECs. The role of pericytes is not only to influence the process of angiogenesis but also to induce the endothelium differentiation towards brain-specific ECs. The influence of astrocytes is visible in increased expression of transport proteins, TJ proteins and a decrease in permeability. 

#### 7.4.6. Microfluidic Systems

Microfluidic models are by far the most promising direction in the evolution of experimental methods in the context of pharmacological tests. Their name comes from the small amounts of reagents for a single model; moreover, because of their small size, the number of cells required for this model is lower than in the DIV-BBB model.

#### 7.4.7. µBBB Model

**Figure 9:**
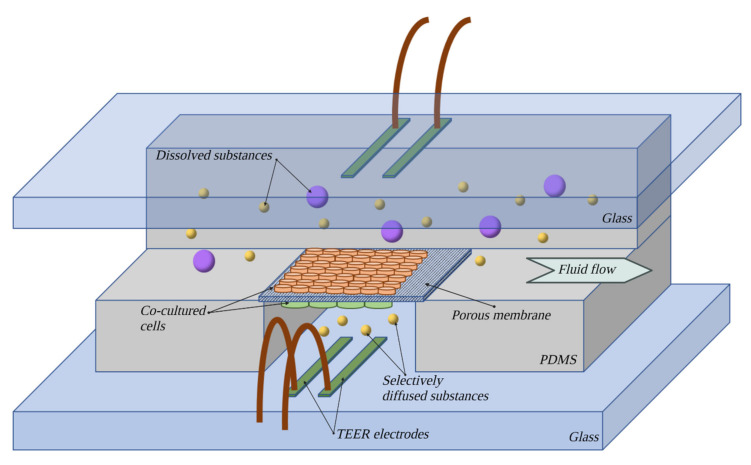
µBBB model. Created with BioRender.com (accessed on 22 February 2023).

The first model of this group is known as the microfluidic blood–brain barrier, µBBB. It is built from four layers of a transparent polymer, polydimethylsalixide (PDMS), enveloped in glass, allowing for visual control of the culture (as shown in Figure 9). Electrodes for real-time TEER measurement are also suspended in PDMS. Two channels formed by PDMS run perpendicularly to each other with a porous membrane set where they briefly merge. On the top side of the membrane, ECs are cultured, while the bottom side holds the astrocytes. This model has undergone modifications since it was first established, the most notable of which was shrinking the surface of contact between channels, which allowed for lowering the number of required cells [76]. This model exhibits TEER values ten times higher than an analogical coculture in the static model [68]. Further advantages are relatively low cost, simplicity of construction and TEER measurement in real time. Notable is also the possibility of strict control over the parameters of the medium and its flow in both channels. High repeatability can also be attained when employing cell lines. The model allows for more precise studies on cocultures influenced by shear stress than DIV-BBB. It has proven useful as a tool for research on inflammatory mechanisms, barrier regeneration after exposure to histamine—a substance known for its BBB-disruptive properties [123]—and migration of cells [117].

The limitation of this model is exhibiting shear stress values much lower than *in vivo*, which can project negatively on parameters induced by this factor [68]. Additionally, for the time being, there are no models of this sort based on human cell lines.

#### 7.4.8. Model BBB-on-a-Chip

Another model called BBB-on-a-chip is a representative of a wide *in vitro* modeling branch, known as organs on chips. It is an immensely interesting and promising group of models and methods, based on developing compact models the size of a computer chip (hence their name) that are easy to build and use. The advantage of these models is the possibility of constructing *in vitro* models of complete organs, along with vasculature, as tools for medical and pharmacological testing. Since 2010, when the first lung-on-a-chip model was developed, other models have also been designed, such as heart, liver, kidney, bone marrow and BBB models [124]. The BBB-on-a-chip is by far the smallest model of this structure; to some degree, it is also an answer to some limitations of previous dynamic models, even more so than µBBB.

**Figure 10:**
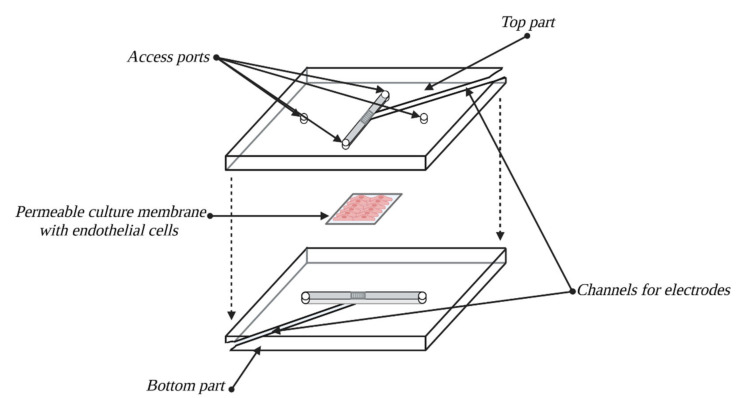
BBB-on-a-chip model. Created with BioRender.com (accessed on 22 February 2023).

The schematic build of this model is shown in Figure 10. The culture chamber is built from two transparent layers of PDMS, with engraved grooves that form flow channels for the medium and access path for TEER electrodes. The zone of channel intersection has been shrunk in comparison to previous models, which enables even smaller amounts of cells and reagents to be used [42,125]. This model has been developed based on the human cell line hCMEC/D3 [126], which contributes to its high scientific value. Additional advantages are, as before, the simplicity of building and handling and TEER measures that can be conducted in real time [127]. An improvement in comparison with µBBB is attaining shear stress values at levels corresponding with *in vivo* conditions. Originally, this model’s limitation was the employment of EC monoculture, which restricted its use by omitting the influence of EC interactions with astrocytes and pericytes. However, since the first chip, an increasing number of advanced chips have been developed, e.g., based on multicultures, where ECs are cultured on the top side of the membrane while cocultures of astrocytes and pericytes are placed on the bottom side [128,129]. For the time being, it seems that these models will play crucial roles in HTS testing.

#### 7.4.9. NVU-on-a-Chip

One of the most complex models of the BBB is NVU-on-a-chip. This model differs from the ones mentioned before because it consists of two modules. The first module, the neural module, holds cultures of neurons, astrocytes and microglia. Second, the vascular module is the environment for EC culture. After culturing, two modules are put together, making one piece, divided by a semipermeable membrane. Next, the construct is enveloped in a layer of transparent PDMS. Culturing two modules separately is dictated by differing optimum culture times; ECs reach confluence after 2–3 days, while the neural module needs 7–10 days [130].

This model is utilized in a complex study on the interactions between components of the NVU (i.a., signaling between them), cerebrospinal fluid [68], NVU changes in response to pathological situations such as brain damage, stroke or Alzheimer’s disease [131], research on the influence of TNF-α on barrier permeability.

The main advantage of this model is its complexity in NVU imaging, and thanks to the presence of all its components that contribute to the emergence of the BBB phenotype, it can be used to attain a highly specialized BBB *in vitro* [130]. Another benefit is high potential in HTS testing under conditions of further development of this model. For the time being, it is burdened with a few limitations, e.g., problems with implementing shear stress in values corresponding to the *in vivo* situation, lack of access to the neural module once the whole model has been assembled, and lack of measuring TEER in real time [68]. However, it seems just a matter of time before these problems are solved and NVU-on-a-chip evolves toward a more advanced model and becomes a powerful tool for research on NVU functioning, the influence of drugs, pathogens, neurotropic factors and, at the same time, the most precise imitation of the NVU and BBB, taking into account a wide spectrum of physiological, biomechanical and morphological aspects [132].

#### 7.4.10. Synthetic Microvasculature of the BBB

**Figure 11:**
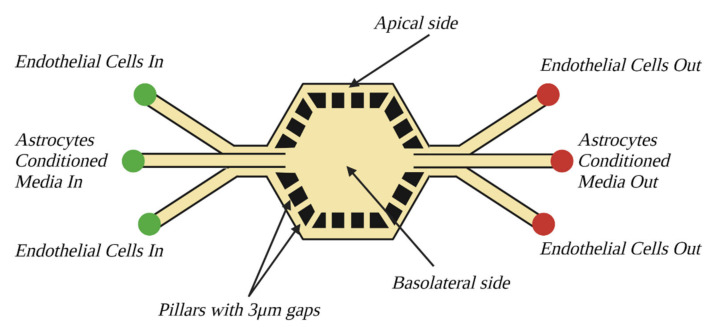
Synthetic microvasculature of the blood–brain barrier (SyM-BBB) model. Created with BioRender.com (accessed on 22 February 2023).

The last model discussed in the context of microfluidic systems is the Synthetic Microvasculature of the Blood–Brain Barrier (SyM-BBB). This model also employs a compact chip made of PDMS; however, it is significantly different from previously discussed models. The main difference is moving from a vertical arrangement; chambers representing luminal and abluminal sides are placed side by side next to each other. The second difference is the resignation of the porous membrane present in previous models, as it was replaced with channels connecting two chambers. The channel diameter is 3 µm, which is a value specified based on pore size in Transwell inserts [68]. On the luminal side, which envelopes the abluminal side, ECs are cultured. Originally, this model was developed based on the RBE4 cell line. The abluminal side carries culture medium conditioned by astrocyte culture. Each chamber is equipped with separate access ports that allow for perfusion and strict control over the culture environment and applied reagents (see Figure 11).

This model is characterized by permeability values far lower than those achieved with Transwell systems and high levels of expression of claudin-1, ZO-1, transport pump proteins and glycoprotein-P [133], which shows that it can be an interesting alternative to semipermeable membranes. The limitations of the original model’s project are the inability to conduct TEER measurements, lack of interactions between cellular components and the relatively large size of BBB emergence, which are inadequate to *in vivo* conditions [68,85]. Nonetheless, this model could potentially be utilized in pharmacological and medical studies.

Another notable model is a low-permeability microfluidic platform described by Noo Li Jeon group. Its most interesting feature is two channels that represent vascular and neuronal domains. The channels allow for providing both ECs and brain cells (the ACs and the neurons were used in this model; however, use of other types of brain cells could be considered) with appropriate conditions. This approach makes both vascular and neuronal sides of the BBB easily accessible for independent treatment [134].

In summary, dynamic models are a promising and constantly evolving branch of BBB modeling methods. They allow for observation of many functional aspects of the BBB that are often impossible to study on static models, therefore mimicking the BBB in a far more precise manner. Their main limitation, especially in the case of microfluidic models, is their restricted accessibility, since many of them are still in the experimental phase; hence, they lack usage protocols. Therefore, utilizing them can be problematic, particularly for laboratories without bioengineering facilities. In reality, it enforces close cooperation with certain model creators. In spite of their potential for pharmacological studies, in most cases they do not fully meet the HTS requirements, which leads to unrelenting use of static models. On the other hand, the advantage of dynamic models over static models is without a doubt their greater usefulness in studies on BBB functions and characteristics. Implementation of the physiological and biomechanical factors that these models offer is perhaps the best way to achieve the most complex model, allowing not only studying every single part of the BBB but also and foremost a way of researching synergic and multidirectional dependencies that connect these components.

## 8. Summary

Although research on the BBB has been conducted for many years, many aspects of its formation in organisms, its significance for the proper functioning of the CNS, and dependencies between its components and the rest of the NVU remain unexplained. From a practical medical point of view, the biggest problem thus far is transport via the barrier that often eliminates the possibility of CNS-targeted therapy. Equally important are issues associated with maintaining the proper functioning of the barrier and its regeneration properties in pathological states, e.g., Alzheimer’s disease, cancer or stroke. Considering the crucial importance of these matters for defeating many diseases that torment our civilization and are natural for all humans’ pursuit of knowledge, in upcoming years we can expect fantastic discoveries and accomplishments in this field.

As presented in the above work, *in vitro* methods are invaluable tools utilized in research on the BBB, and their significance will increase with the development of new, more advanced models. Although designing a perfect model still seems far away, artificial barriers can only be as perfect as our knowledge is advanced. Even now, many developed models make great research tools, even if the results attained with them will have to be later evaluated with *in vivo* tests. The idea of *in vitro* testing fits perfectly into bioethical narratives, as it reduces the number of animals for testing and generally reduces the number of tests run on living organisms. Technological advances will surely allow for large-scale testing and research without moral dilemmas and without being forced to put human well-being over that of animals.

Looking back on accomplishments of *in vitro* modeling thus far, further evolution can be predicted to some degree. On the one hand, arising models will pursue HTS testing standards, and on the other hand, the most accurate imaging of barriers and their functions will be pursued. It could seem that these two paths lead in opposite directions, and it is clear that to achieve perfection in mimicking the BBB, increasingly complex systems are being developed, which are naturally harder and harder to utilize and increasingly costly. For HTS testing, on the other hand, they should be as cheap, simple and repeatable as possible. In reality, it can be observed that models that are too complicated, complex and expensive at first undergo evolution toward being more compact and useful for pharmacological and medical testing. It should be expected that more advanced microfluidic models will mainly arise, most likely based on multicultures and employing both a wide spectrum of cellular and noncellular components as well as biomechanical factors. Another promising direction seems to be 3D ECM-gel cultures, due to their ability to mimic BBB development, and the emergence of interactions between components. To achieve the best models possible, especially ECM-gel models, it is crucial to precisely study and recreate the matrix’s structure and composition. The need for strict usage procedures appears obvious, as does the standardizing of culture conditions and measuring methods. Only such an approach allows for attaining results that are not only reliable and repeatable but also comparable between labs all over the world. Taking the above aspects into consideration, it can be concluded that the upcoming years will see a dynamic and intense evolution of both *in vitro* modeling methods and general knowledge on the blood–brain barrier and central nervous system. Without any doubt, it will be a process worth tracking and, even more, worth taking part in.

## Data Availability

Not applicable.

## Abbreviations

- AJ—Adherens junctions
- BBB—Blood–brain barrier
- BM—Basement membrane
- CNS—Central nervous system
- EC—Endothelial cells
- ECM—Extracellular matrix
- HTS—High-throughput screening
- NVU—Neurovascular unit
- TEER—Transendothelial electrical resistance
- TJ—Tight junctions

## References

[1] Abbott N.J., Rönnbäck L., Hansson E. (2006). Astrocyte-endothelial interactions at the blood-brain barrier. Nat. Rev. Neurosci..

[2] Yuan Y., Sun J., Dong Q., Cui M. (2023). Blood-brain barrier endothelial cells in neurodegenerative diseases: Signals from the “barrier”. Front. Neurosci..

[3] Lee S., Chung M., Lee S.R., Jeon N. (2019). 3D brain angiogenesis model to reconstitute functional human blood-brain barrier *in vitro*. Biotechnol. Bioeng..

[4] Memis I., Mittal R., Furar E., White I., Eshraghi R.S., Mittal J., Eshraghi A.A. (2022). Altered Blood Brain Barrier Permeability and Oxidative Stress in *Cntnap2* Knockout Rat Model. J. Clin. Med..

[5] Najjar S., Pahlajani S., De Sanctis V., Stern J.N.H., Najjar A., Chong D. (2017). Neurovascular Unit Dysfunction and Blood-Brain Barrier Hyperpermeability Contribute to Schizophrenia Neurobiology: A Theoretical Integration of Clinical and Experimental Evidence. Front. Psychiatry.

[6] Lazarovici P., Li M., Perets A., Mondrinos M.J., Lecht S., Koharski C.D., Bidez P.R., Finck C.M., Lelkes P.I., Marx U., Sandig V. (2007). Intelligent Biomatrices and Engineered Tissue Constructs: In-Vitro Models for Drug Discovery and Toxicity Testing. Drug Testing In Vitro Breakthroughs and Trends in Cell Culture Technology.

[7] Koper-Lenkiewicz O.M., Kamińska J., Lewoniewska S., Wilińska E. (2018). Rola bariery krew-płyn mózgowo-rdzeniowy i bariery krew mózg w utrzymaniu homeostazy ośrodkowego układu nerwowego. Pol. Przegl. Neurol..

[8] Barichello T., Collodel A., Hasbun R., Morales R., Barichello T. (2019). An Overview of the Blood-Brain Barrier. Blood Brain Barrier.

[9] Wilhelm I., Fazakas C., Krizbai I.A. (2011). *In vitro* models of the blood-brain barrier. Acta Neurobiol. Exp..

[10] González-Mariscal L., Betanzos A., Nava P., Jaramillo B.E. (2003). Tight junction proteins. Prog. Biophys. Mol. Biol..

[11] Furuse M., Hirase T., Itoh M., Nagafuchi A., Yonemura S., Tsukita S. (1993). Occludin: A novel integral membrane protein localizing at tight junctions. J. Cell Biol..

[12] Sugiyama S., Sasaki T., Tanaka H., Yan H., Ikegami T., Kanki H., Nishiyama K., Beck G., Gon Y., Okazaki S. (2023). The tight junction protein occludin modulates blood-brain barrier integrity and neurological function after ischemic stroke in mice. Sci. Res..

[13] Martìn-Padura I., Lostaglio S., Schneemann M., Williams L., Romano M., Fruscella P., Panzeri C., Stoppacciaro A., Ruco L., Villa A. (1998). Junctional adhesion molecule, a novel member of the immunoglobulin superfamily that distributes at intercellular junctions and modulates monocyte transmigration. J. Cell Biol..

[14] Schulze C., Firth J.A. (1993). Immunohistochemical localization of adherens junction components in blood-brain barrier microvessels of the rat. J. Cell Sci..

[15] Kopec B.M., Ulapane K.R., Moral M.E.G., Siahaan T.J., Barichello T. (2019). Methods of Delivering Molecules Through the Blood-Brain Barrier for Brain Diagnostics and Therapeutics. Blood Brain Barrier.

[16] Garrod D., Chidgey M. (2008). Desmosome structure, composition and function. Biochim. Biophys. Acta.

[17] Bushong E.A., Martone M.E., Jones Y.Z., Ellisman M.H. (2002). Protoplasmic astrocytes in CA1 stratum radiatum occupy separate anatomical domains. J. Neurosci..

[18] Dehouck M.P., Méresse S., Delorme P., Fruchart J.C., Cecchelli R. (1990). An easier, reproducible, and mass-production method to study the blood-brain barrier *in vitro*. J. Neurochem..

[19] Schinkel A.H. (1999). P-Glycoprotein, a gatekeeper in the blood-brain barrier. Adv. Drug Deliv. Rev..

[20] Haseloff R.F., Blasig I.E., Bauer H.C., Bauer H. (2005). In search of the astrocytic factor(s) modulating blood-brain barrier functions in brain capillary endothelial cells *in vitro*. Cell. Mol. Neurobiol..

[21] Gotoh M., Miyamoto Y., Ikeshima-Kataoka H. (2023). Astrocytic Neuroimmunological Roles Interacting with Microglial Cells in Neurodegenerative Diseases. Int. J. Mol. Sci..

[22] Ramsauer M., Krause D., Dermietzel R. (2002). Angiogenesis of the blood-brain barrier *in vitro* and the function of cerebral pericytes. FASEB J..

[23] Mi H., Haeberle H., Barres B.A. (2001). Induction of astrocyte differentiation by endothelial cells. J. Neurosci..

[24] Schroeter M.L., Mertsch K., Giese H., Müller S., Sporbert A., Hickel B., Blasig I.E. (1999). Astrocytes enhance radical defence in capillary endothelial cells constituting the blood-brain barrier. FEBS Lett..

[25] Yamazaki T., Mukouyama Y.S. (2018). Tissue Specific Origin, Development, and Pathological Perspectives of Pericytes. Front. Cardiovasc. Med..

[26] McConnell H.L., Kersch C.N., Woltjer R.L., Neuwelt E.A. (2017). The Translational Significance of the Neurovascular Unit. J. Biol. Chem..

[27] Dohgu S., Takata F., Yamauchi A., Nakagawa S., Egawa T., Naito M., Tsuruo T., Sawada Y., Niwa M., Kataoka Y. (2005). Brain pericytes contribute to the induction and up-regulation of blood-brain barrier functions through transforming growth factor-beta production. Brain Res.

[28] Sá-Pereira I., Brites D., Brito M.A. (2012). Neurovascular unit: A focus on pericytes. Mol. Neurobiol..

[29] Shimizu F., Sano Y., Maeda T., Abe M.A., Nakayama H., Takahashi R., Ueda M., Ohtsuki S., Terasaki T., Obinata M. (2008). Peripheral nerve pericytes originating from the blood-nerve barrier expresses tight junctional molecules and transporters as barrier-forming cells. J. Cell. Physiol..

[30] Fisher M. (2009). Pericyte signaling in the neurovascular unit. Stroke.

[31] Alarcon-Martinez L., Yemisci M., Dalkara T. (2021). Pericyte morphology and function. Histol. Histopathol..

[32] Chaulagain B., Gothwal A., Lamptey R.N.L., Trivedi R., Mahanta A.K., Layek B., Singh J. (2023). Experimental Models of *In Vitro* Blood-Brain Barrier for CNS Drug Delivery: An Evolutionary Perspective. Int. J. Mol. Sci..

[33] Dore-Duffy P., Katychev A., Wang X., Van Buren E. (2006). CNS microvascular pericytes exhibit multipotential stem cell activity. J. Cereb. Blood Flow Metab..

[34] Nakagomi T., Kubo S., Nakano-Doi A., Sakuma R., Lu S., Narita A., Kawahara M., Taguchi A., Matsuyama T. (2015). Brain vascular pericytes following ischemia have multipotential stem cell activity to differentiate into neural and vascular lineage cells. Stem Cells.

[35] Stratman A.N., Davis G.E. (2012). Endothelial cell-pericyte interactions stimulate basement membrane matrix assembly: Influence on vascular tube remodeling, maturation, and stabilization. Microsc. Microanal..

[36] Bell A.H., Miller S.L., Castillo-Melendez M., Malhotra A. (2020). The Neurovascular Unit: Effects of Brain Insults During the Perinatal Period. Front. Neurosci..

[37] Yurchenco P.D., Patton B.L. (2009). Developmental and pathogenic mechanisms of basement membrane assembly. Curr. Pharm. Des..

[38] Cardoso F.L., Brites D., Brito M.A. (2010). Looking at the blood-brain barrier: Molecular anatomy and possible investigation approaches. Brain Res. Rev..

[39] Tontsch U., Bauer H.C. (1991). Glial cells and neurons induce blood-brain barrier related enzymes in cultured cerebral endothelial cells. Brain Res..

[40] Stewart P.A., Wiley M.J. (1981). Developing nervous tissue induces formation of blood-brain barrier characteristics in invading endothelial cells: A study using quail—Chick transplantation chimeras. Dev. Biol..

[41] Pulido R.S., Munji R.N., Chan T.C., Quirk C.R., Weiner G.A., Weger B.D., Rossi M.J., Elmsaouri S., Malfavon M., Deng A. (2020). Neuronal Activity Regulates Blood-Brain Barrier Efflux Transport through Endothelial Circadian Genes. Neuron.

[42] Guarino V., Zizzari A., Bianco M., Gigli G., Moroni L., Arima V. (2023). Advancements in modelling human blood brain-barrier on a chip. Biofabrication.

[43] Stankovic N.D., Teodorczyk M., Ploen R., Zipp F., Schmidt M.H.H. (2016). Microglia–blood vessel interactions: A double-edged sword in brain pathologies. Acta Neuropathol..

[44] Hickey W.F., Kimura H. (1988). Perivascular microglial cells of the CNS are bone marrow-derived and present antigen *in vivo*. Science.

[45] Hawkes C.A., McLaurin J. (2009). Selective targeting of perivascular macrophages for clearance of beta-amyloid in cerebral amyloid angiopathy. Proc. Natl. Acad. Sci. USA.

[46] Abbott N.J., Romero I.A. (1996). Transporting therapeutics across the blood-brain barrier. Mol. Med. Today.

[47] Lalatsa A., Butt A.M., Kesharwani P., Gupta U. (2018). Physiology of the Blood Brain Barrier and Mechanisms of Transport Across the BBB. Nanotechnology-Based Targeted Drug Delivery Systems for Brain Tumors.

[48] Carruthers A., DeZutter J., Ganguly A., Devaskar S.U. (2009). Will the original glucose transporter isoform please stand up!. Am. J. Physiol. Endocrinol. Metab..

[49] Wade L.A., Katzman R. (1975). Synthetic amino acids and the nature of L-DOPA transport at the blood-brain barrier. J. Neurochem..

[50] Morris M.E., Rodriguez-Cruz V., Felmlee M.A. (2017). SLC and ABC Transporters: Expression, Localization, and Species Differences at the Blood-Brain and the Blood-Cerebrospinal Fluid Barriers. AAPS J..

[51] Bickel U., Yoshikawa T., Pardridge W.M. (2001). Delivery of peptides and proteins through the blood-brain barrier. Adv. Drug Deliv. Rev..

[52] Broadwell R.D., Balin B.J., Salcman M. (1988). Transcytotic pathway for blood-borne protein through the blood-brain barrier. Proc. Natl. Acad. Sci. USA.

[53] Vorbrodt A.W. (1989). Ultracytochemical characterization of anionic sites in the wall of brain capillaries. J. Neurocytol..

[54] Jiang T., Olson E.S., Nguyen Q.T., Roy M., Jennings P.A., Tsien R.Y. (2004). Tumor imaging by means of proteolytic activation of cell-penetrating peptides. Proc. Natl. Acad. Sci. USA.

[55] Elbrecht D.H., Long C.J., Hickman J.J. (2016). Transepithelial/endothelial Electrical Resistance (TEER) theory and applications for microfluidic body-on-a-chip devices. J. Rare Dis. Res. Treat..

[56] Szulcek R., Bogaard H.J., van Nieuw Amerongen G.P. (2014). Electric cell-substrate impedance sensing for the quantification of endothelial proliferation, barrier function, and motility. J. Vis. Exp..

[57] Matter K., Balda M.S. (2003). Functional analysis of tight junctions. Methods.

[58] Blume L.F., Denker M., Gieseler F., Kunze T. (2010). Temperature corrected transepithelial electrical resistance (TEER) measurement to quantify rapid changes in paracellular permeability. Pharmazie.

[59] Lu S., Gough A.W., Bobrowski W.F., Stewart B.H. (1996). Transport properties are not altered across Caco-2 cells with heightened TEER despite underlying physiological and ultrastructural changes. J. Pharm. Sci..

[60] Rubin L.L., Hall D.E., Porter S., Barbu K., Cannon C., Horner H.C., Janatpour M., Liaw C.W., Manning K., Morales J. (1991). A cell culture model of the blood-brain barrier. J. Cell Biol..

[61] Wang Y., Wang N., Cai B., Wang G.Y., Li J., Piao X.X. (2015). *In vitro* model of the blood-brain barrier established by co-culture of primary cerebral microvascular endothelial and astrocyte cells. Neural Regen. Res..

[62] Lauschke K., Frederiksen L., Hall V.J. (2017). Paving the Way Toward Complex Blood-Brain Barrier Models Using Pluripotent Stem Cells. Stem Cells Dev..

[63] Srinivasan B., Kolli A.R., Esch M.B., Abaci H.E., Shuler M.L., Hickman J.J. (2015). TEER measurement techniques for *in vitro* barrier model systems. J. Lab. Autom..

[64] Severs N.J. (2007). Freeze-fracture electron microscopy. Nat. Protoc..

[65] Lane N.J., Abbott N.J. (1992). Freeze-fracture evidence for a novel restricting junction at the blood-brain barrier of the cuttlefish Sepia officinalis. J. Neurocytol..

[66] Mullier A., Bouret S.G., Prevot V., Dehouck B. (2010). Differential distribution of tight junction proteins suggests a role for tanycytes in blood-hypothalamus barrier regulation in the adult mouse brain. J. Comp. Neurol..

[67] Meyer J., Mischeck U., Veyhl M., Henzel K., Galla H.J. (1990). Blood-brain barrier characteristic enzymatic properties in cultured brain capillary endothelial cells. Brain Res..

[68] Palmiotti C.A., Prasad S., Naik P., Abul K.M., Sajja R.K., Achyuta A.H., Cucullo L. (2014). *In vitro* cerebrovascular modeling in the 21st century: Current and prospective technologies. Pharm. Res..

[69] Wilhelm I., Krizbai I.A. (2014). *In vitro* models of the blood-brain barrier for the study of drug delivery to the brain. Mol. Pharm..

[70] Pidgeon C., Ong S., Liu H., Qiu X., Pidgeon M., Dantzig A.H., Munroe J., Hornback W.J., Kasher J.S., Glunz L. (1995). IAM chromatography: An *in vitro* screen for predicting drug membrane permeability. J. Med. Chem..

[71] Kansy M., Senner F., Gubernator K. (1998). Physicochemical high throughput screening: Parallel artificial membrane permeation assay in the description of passive absorption processes. J. Med. Chem..

[72] Avdeef A. (2005). The rise of PAMPA. Expert Opin. Drug Metab. Toxicol..

[73] Ecker G.F., Noe C.R. (2004). In silico prediction models for blood-brain barrier permeation. Curr. Med. Chem..

[74] Peterson D.R., Hawkins R.A., Nag S. (2003). Transport Studies Using Membrane Vesicles. The Blood–Brain Barrier: Biology and Research Protocols.

[75] Glavinas H., Méhn D., Jani M., Oosterhuis B., Herédi-Szabó K., Krajcsi P. (2008). Utilization of membrane vesicle preparations to study drug-ABC transporter interactions. Expert Opin. Drug Metab. Toxicol..

[76] Bagchi S., Chhibber T., Lahooti B., Verma A., Borse V., Jayant R.D. (2019). In-vitro blood-brain barrier models for drug screening and permeation studies: An overview. Drug Des. Dev. Ther..

[77] Hellinger E., Veszelka S., Tóth A.E., Walter F., Kittel A., Bakk M.L., Tihanyi K., Háda V., Nakagawa S., Duy T.D. (2012). Comparison of brain capillary endothelial cell-based and epithelial (MDCK-MDR1, Caco-2, and VB-Caco-2) cell-based surrogate blood-brain barrier penetration models. Eur. J. Pharm. Biopharm..

[78] Lundquist S., Renftel M., Brillault J., Fenart L., Cecchelli R., Dehouck M.P. (2002). Prediction of drug transport through the blood-brain barrier *in vivo*: A comparison between two *in vitro* cell models. Pharm. Res..

[79] Wang Q., Rager J.D., Weinstein K., Kardos P.S., Dobson G.L., Li J., Hidalgo I.J. (2005). Evaluation of the MDR-MDCK cell line as a permeability screen for the blood-brain barrier. Int. J. Pharm..

[80] Xie J., Lei C., Hu Y., Gay G.K., Bin Jamali N.H., Wang C.H. (2010). Nanoparticulate formulations for paclitaxel delivery across MDCK cell monolayer. Curr. Pharm. Des..

[81] Man S., Ubogu E.E., Williams K.A., Tucky B., Callahan M.K., Ransohoff R.M. (2008). Human brain microvascular endothelial cells and umbilical vein endothelial cells differentially facilitate leukocyte recruitment and utilize chemokines for T cell migration. Clin. Dev. Immunol..

[82] Langford D., Hurford R., Hashimoto M., Digicaylioglu M., Masliah E. (2005). Signalling crosstalk in FGF2-mediated protection of endothelial cells from HIV-gp120. BMC Neurosci..

[83] Choi K.D., Yu J., Smuga-Otto K., Salvagiotto G., Rehrauer W., Vodyanik M., Thomson J., Slukvin I. (2009). Hematopoietic and endothelial differentiation of human induced pluripotent stem cells. Stem Cells.

[84] Lippmann E.S., Azarin S.M., Kay J.E., Nessler R.A., Wilson H.K., Al-Ahmad A., Palecek S.P., Shusta E.V. (2012). Derivation of blood-brain barrier endothelial cells from human pluripotent stem cells. Nat. Biotechnol..

[85] Gomes M.J., Mendes B., Martins S., Sarmento B., Sarmento B. (2016). Cell-based *in vitro* models for studying blood–brain barrier (BBB) permeability. Concepts and Models for Drug Permeability Studies.

[86] Helms H.C., Abbott N.J., Burek M., Cecchelli R., Couraud P.O., Deli M.A., Förster C., Galla H.J., Romero I.A., Shusta E.V. (2016). *In vitro* models of the blood-brain barrier: An overview of commonly used brain endothelial cell culture models and guidelines for their use. J. Cereb. Blood Flow Metab..

[87] Silbergeld D.L., Ali-Osman F. (1991). Isolation and characterization of microvessels from normal brain and brain tumors. J. Neurooncol..

[88] Miller D.S., Graeff C., Droulle L., Fricker S., Fricker G. (2002). Xenobiotic efflux pumps in isolated fish brain capillaries. Am. J. Physiol. Regul. Integr. Comp. Physiol..

[89] Catalán R.E., Martínez A.M., Aragonés M.D., Hernández F., Diáz G. (1996). Endothelin stimulates protein phosphorylation in blood-brain barrier. Biochem. Biophys. Res. Commun..

[90] Durk M.R., Han K., Chow E.C., Ahrens R., Henderson J.T., Fraser P.E., Pang K.S. (2014). 1α,25-Dihydroxyvitamin D3 reduces cerebral amyloid-β accumulation and improves cognition in mouse models of Alzheimer’s disease. J. Neurosci..

[91] Salvador E., Köppl T., Hörmann J., Schönhärl S., Bugaeva P., Kessler A.F., Burek M., Ernestus R.-I., Löhr M., Hagemann C. (2023). Tumor Treating Fields (TTFields) Induce Cell Junction Alterations in a Human 3D *In Vitro* Model of the Blood-Brain Barrier. Pharmaceutics.

[92] Tóth A., Veszelka S., Nakagawa S., Niwa M., Deli M.A. (2011). Patented *in vitro* blood-brain barrier models in CNS drug discovery. Recent Pat. CNS Drug Discov..

[93] Weksler B.B., Subileau E.A., Perrière N., Charneau P., Holloway K., Leveque M., Tricoire-Leignel H., Nicotra A., Bourdoulous S., Turowski P. (2005). Blood-brain barrier-specific properties of a human adult brain endothelial cell line. FASEB J..

[94] Kaisar M.A., Sajja R.K., Prasad S., Abhyankar V.V., Liles T., Cucullo L. (2017). New experimental models of the blood-brain barrier for CNS drug discovery. Expert Opin. Drug Discov..

[95] Lippmann E.S., Azarin S.M., Palecek S.P., Shusta E.V. (2020). Commentary on human pluripotent stem cell-based blood-brain barrier models. Fluids Barriers CNS.

[96] Girard S.D., Julien-Gau I., Molino Y., Combes B.F., Greetham L., Khrestchatisky M., Nivet E. (2023). High and low permeability of human pluripotent stem cell-derived blood-brain barrier models depend on epithelial or endothelial features. FASEB J..

[97] Singh N.R., Gromnicova R., Brachner A., Kraev I., Romero I.A., Neuhaus W., Male D. (2023). A hydrogel model of the human blood-brain barrier using differentiated stem cells. PLoS ONE.

[98] Ogunshola O.O. (2011). *in vitro* modeling of the blood-brain barrier: Simplicity versus complexity. Curr. Pharm. Des..

[99] Patabendige A., Skinner R.A., Abbott N.J. (2012). Establishment of a simplified *in vitro* porcine blood-brain barrier model with high transendothelial electrical resistance. Brain Res..

[100] Cantrill C.A., Skinner R.A., Rothwell N.J., Penny J.I. (2012). An immortalised astrocyte cell line maintains the *in vivo* phenotype of a primary porcine *in vitro* blood-brain barrier model. Brain Res..

[101] Burek M., Forster C.Y., Barichello T. (2019). Culturing of Rodent Brain Microvascular Endothelial Cells for *In Vitro*. Blood Brain Barrier.

[102] Smith M., Omidi Y., Gumbleton M. (2007). Primary porcine brain microvascular endothelial cells: Biochemical and functional characterisation as a model for drug transport and targeting. J. Drug Target..

[103] Park J.S., Choe K., Khan A., Jo M.H., Park H.Y., Kang M.H., Park T.J., Kim M.O. (2023). Establishing Co-Culture Blood-Brain Barrier Models for Different Neurodegeneration Conditions to Understand Its Effect on BBB Integrity. Int. J. Mol. Sci..

[104] Schiera G., Sala S., Gallo A., Raffa M.P., Pitarresi G.L., Savettieri G., Di Liegro I. (2005). Permeability properties of a three-cell type *in vitro* model of blood-brain barrier. J. Cell. Mol. Med..

[105] Boghdeh N.A., Risner K.H., Barrera M.D., Britt C.M., Schaffer D.K., Alem F., Brown J.A., Wikswo J.P., Narayanan A. (2022). Application of a Human Blood Brain Barrier Organ-on-a-Chip Model to Evaluate Small Molecule Effectiveness against Venezuelan Equine Encephalitis Virus. Viruses.

[106] Boveri M., Berezowski V., Price A., Slupek S., Lenfant A.M., Benaud C., Hartung T., Cecchelli R., Prieto P., Dehouck M.P. (2005). Induction of blood-brain barrier properties in cultured brain capillary endothelial cells: Comparison between primary glial cells and C6 cell line. Glia.

[107] Khodarev N.N., Yu J., Labay E., Darga T., Brown C.K., Mauceri H.J., Yassari R., Gupta N., Weichselbaum R.R. (2003). Tumour-endothelium interactions in co-culture: Coordinated changes of gene expression profiles and phenotypic properties of endothelial cells. J. Cell. Sci..

[108] Song Y., Cai X., Du D., Dutta P., Lin Y. (2019). Comparison of Blood-Brain Barrier Models for *in vitro* Biological Analysis: One Cell Type vs Three Cell Types. ACS Appl. Bio Mater..

[109] Rochfort K.D., Cummins P.M., Barichello T. (2019). Demonstrating the Beneficial Influence of Shear Stress on Brain Microvascular Endothelial Cell Phenotype. Blood Brain Barrier.

[110] Tarbell J.M. (2010). Shear stress and the endothelial transport barrier. Cardiovasc. Res..

[111] Cucullo L., Hossain M., Puvenna V., Marchi N., Janigro D. (2011). The role of shear stress in Blood-Brain Barrier endothelial physiology. BMC Neurosci..

[112] Bussolari S.R., Dewey C.F., Gimbrone M.A. (1982). Apparatus for subjecting living cells to fluid shear stress. Rev. Sci. Instrum..

[113] Man S., Tucky B., Cotleur A., Drazba J., Takeshita Y., Ransohoff R.M. (2012). CXCL12-induced monocyte-endothelial interactions promote lymphocyte transmigration across an *in vitro* blood-brain barrier. Sci. Transl. Med..

[114] Wong A.K., Llanos P., Boroda N., Rosenberg S.R., Rabbany S.Y. (2016). A Parallel-Plate Flow Chamber for Mechanical Characterization of Endothelial Cells Exposed to Laminar Shear Stress. Cell. Mol. Bioeng..

[115] Kemeny S.F., Figueroa D.S., Clyne A.M. (2013). Hypo- and hyperglycemia impair endothelial cell actin alignment and nitric oxide synthase activation in response to shear stress. PLoS ONE.

[116] Parkinson F.E., Friesen J., Krizanac-Bengez L., Janigro D. (2003). Use of a three-dimensional *in vitro* model of the rat blood-brain barrier to assay nucleoside efflux from brain. Brain Res..

[117] Naik P., Cucullo L. (2012). *In vitro* blood-brain barrier models: Current and perspective technologies. J. Pharm. Sci..

[118] Kaisar M.A., Abhyankar V.V., Cucullo L., Barichello T. (2019). *In Vitro* BBB Models: Working with Static Platforms and Microfluidic Systems. Blood Brain Barrier.

[119] Cucullo L., Couraud P.O., Weksler B., Romero I.A., Hossain M., Rapp E., Janigro D. (2008). Immortalized human brain endothelial cells and flow-based vascular modeling: A marriage of convenience for rational neurovascular studies. J. Cereb. Blood Flow Metab..

[120] Nagpal K., Singh S.K., Mishra D.N. (2013). Drug targeting to brain: A systematic approach to study the factors, parameters and approaches for prediction of permeability of drugs across BBB. Expert Opin. Drug Deliv..

[121] Da Silva K., Kumar P., Choonara Y.E., du Toit L.C., Pillay V. (2020). Three-dimensional printing of extracellular matrix (ECM)-mimicking scaffolds: A critical review of the current ECM materials. J. Biomed. Mater. Res. A.

[122] Yim E.K., Leong K.W. (2005). Proliferation and differentiation of human embryonic germ cell derivatives in bioactive polymeric fibrous scaffold. J. Biomater. Sci. Polym. Ed..

[123] Stamatovic S.M., Keep R.F., Andjelkovic A.V. (2008). Brain endothelial cell-cell junctions: How to “open” the blood brain barrier. Curr. Neuropharmacol..

[124] Tang H., Abouleila Y., Si L., Ortega-Prieto A.M., Mummery C.L., Ingber D.E., Mashaghi A. (2020). Human Organs-on-Chips for Virology. Trends Microbiol..

[125] Palma-Florez S., López-Canosa A., Moralez-Zavala F., Castaño O., Kogan M.J., Samitier J., Lagunas A., Mir M. (2023). BBB-on-a-chip with integrated micro-TEER for permeability evaluation of multi-functionalized gold nanorods against Alzheimer’s disease. J. Nanobiotechnol..

[126] Griep L.M., Wolbers F., de Wagenaar B., ter Braak P.M., Weksler B.B., Romero I.A., Couraud P.O., Vermes I., van der Meer A.D., van den Berg A. (2013). BBB on chip: Microfluidic platform to mechanically and biochemically modulate blood-brain barrier function. Biomed. Microdevices.

[127] Pérez-López A., Isabel Torres-Suárez A., Martín-Sabroso C., Aparicio-Blanco J. (2023). An overview of *in vitro* 3D models of the blood-brain barrier as a tool to predict the *in vivo* permeability of nanomedicines. Adv. Drug Deliv. Rev..

[128] Vatine G.D., Barrile R., Workman M.J., Sances S., Barriga B.K., Rahnama M., Barthakur S., Kasendra M., Lucchesi C., Kerns J. (2019). Human iPSC-Derived Blood-Brain Barrier Chips Enable Disease Modeling and Personalized Medicine Applications. Cell Stem Cell.

[129] Kincses A., Vigh J.P., Petrovszki D., Valkai S., Kocsis A.E., Walter F.R., Lin H.-Y., Jan J.-S., Deli M.A., Dér A. (2023). The Use of Sensors in Blood-Brain Barrier-on-a-Chip Devices: Current Practice and Future Directions. Biosensors.

[130] Achyuta A.K., Conway A.J., Crouse R.B., Bannister E.C., Lee R.N., Katnik C.P., Behensky A.A., Cuevas J., Sundaram S.S. (2013). A modular approach to create a neurovascular unit-on-a-chip. Lab Chip.

[131] Maoz B.M., Herland A., FitzGerald E.A. (2018). A linked organ-on-chip model of the human neurovascular unit reveals the metabolic coupling of endothelial and neuronal cells. Nat. Biotechnol..

[132] Alcendor D.J., Iii F.E.B., Cliffel D.E., Daniels J.S., Ellacott K.L., Goodwin C.R., Hofmeister L.H., Li D., Markov D.A., May J.C. (2013). Neurovascular unit on a chip: Implications for translational applications. Stem Cell Res. Ther..

[133] Prabhakarpandian B., Shen M.C., Nichols J.B., Mills I.R., Sidoryk-Wegrzynowicz M., Aschner M., Pant K. (2013). SyM-BBB: A microfluidic Blood Brain Barrier model. Lab Chip.

[134] Bang S., Lee S.R., Ko J., Son K., Tahk D., Ahn J., Im C., Jeon N.J. (2017). A Low Permeability Microfluidic Blood-Brain Barrier Platform with Direct Contact between Perfusable Vascular Network and Astrocytes. Sci. Rep..

